# Development and multi-cohort validation of a sarcopenia-inflammation index for predicting all-cause mortality in patients with chronic kidney disease

**DOI:** 10.3389/fnut.2026.1852039

**Published:** 2026-08-24

**Authors:** Xiaolong Wang, Jian Yang, Yanjun Liang, Zheyi Dong, Shuang Liang

**Affiliations:** 1 Department of Nephrology, First Medical Center of Chinese PLA General Hospital, Chinese PLA Institute of Nephrology, Beijing, China; 2 Department of Nephrology, Characteristic Medical Center of Chinese People's Armed Police Force, Tianjin, China

**Keywords:** chronic kidney disease, inflammation, mortality, sarcopenia, SI index

## Abstract

**Purpose:**

Sarcopenia and chronic systemic inflammation frequently co-occur in chronic kidney disease (CKD), collectively driving poor prognosis. Yet current clinical practice lacks an integrated metric to quantify their combined prognostic impact. This study aimed to develop and validate a novel Sarcopenia-Inflammation (SI) Index for predicting all-cause mortality in CKD patients.

**Methods:**

This prospective multi-cohort study utilized the C-OPTION cohort (*N* = 539) as both the discovery and primary validation dataset. To assess cross-population generalizability across distinct ethnic groups and healthcare settings, the SI Index was further evaluated in two large population-representative datasets: NHANES (*N* = 1,731) and CHARLS (*N* = 2,625). The SI Index was constructed as a weighted linear combination of sarcopenia status and inflammatory markers derived from multivariate Cox regression coefficients. Predictive performance was evaluated using time-dependent ROC curves, C-index, and AIC/BIC. Incremental prognostic value, 10-fold cross-validation, and subgroup analyses were performed to confirm model robustness.

**Results:**

Among 4,895 CKD patients, multivariate Cox regression identified sarcopenia (HR: 4.582–5.947) and high inflammatory status (HR: 1.43–2.41) as independent mortality determinants with additive effects (interaction *p* > 0.05). The SI Index was established as: 1.522 × Sarcopenia + 1.300 × Abnormal Muscle Traits + 0.880 × High Inflammation. External validation demonstrated superior prognostic discrimination over single-dimension markers, elevating the C-index from 0.623–0.684 (sarcopenia alone) and 0.557–0.584 (inflammation alone) to 0.657–0.741. Incorporating the SI Index into a base clinical model significantly improved C-index from 0.565–0.726 to 0.729–0.768 (all *p* < 0.001), with optimal AIC/BIC. Associations remained robust across subgroups stratified by age, sex, comorbidities, and renal function, and 10-fold cross-validation confirmed model stability.

**Conclusion:**

The SI Index is a potent, reproducible prognostic tool that integrates muscle decline and systemic inflammation to enhance all-cause mortality prediction in CKD, providing a quantitative basis for identifying high-risk patients and guiding personalized intervention strategies.

## Introduction

Chronic kidney disease (CKD) has evolved into a major global public health challenge. Recent epidemiological data estimate the global prevalence of CKD at approximately 10%, affecting over 800 million individuals and ranking it among the leading causes of mortality worldwide (1–3). As CKD progresses, patients frequently encounter a constellation of severe complications, including cardiovascular events, anemia, mineral and bone disorders, and protein-energy wasting (PEW), which collectively drive significant increases in morbidity and mortality (4, 5). Among these complications, sarcopenia—a syndrome characterized by the progressive loss of skeletal muscle mass, strength, and function—has emerged as a critical yet underappreciated determinant of prognosis in the CKD population (6, 7).

The prevalence of sarcopenia in CKD exhibits substantial heterogeneity depending on diagnostic criteria, disease stage, and the study population, ranging broadly from 4 to 63%. Specifically, rates are reported as high as 20–63% in dialysis-dependent patients and 5.9–28.7% in non-dialysis CKD patients (8, 9). This wide variation underscores the urgent need for standardized assessment protocols. Notably, CKD-associated sarcopenia is not merely a consequence of age-dependent degeneration but represents a uremia-driven phenotype of “accelerated aging,” appearing prematurely in relatively young patients (9). Crucially, the adverse prognostic implications of sarcopenia span the entire CKD disease continuum. In patients with end-stage kidney disease (ESKD), sarcopenia is strongly associated with a two-fold increase in hospitalization risk (relative risk [RR] 2.07) (10), a four-fold increase in cardiovascular events (HR 4.33) (11), and a nearly seven-fold increase in all-cause mortality (HR 6.99) (11). Even within the non-dialysis CKD population, sarcopenia has been confirmed as an independent predictor of disease progression, nearly doubling the risk of ESKD (HR 1.98) (9) and tripling the risk of mortality (HR 2.89) (12), highlighting its pivotal value as an early warning signal.

Concurrently, persistent low-grade systemic inflammation is a core feature of CKD pathogenesis. Recently, the Systemic Immune-Inflammation Index (SII)—a composite metric integrating platelet, neutrophil, and lymphocyte counts—has emerged as a superior prognostic marker, capturing the organism’s inflammatory burden more comprehensively than single inflammatory markers (13). Prior studies have established elevated SII as a potent predictor of mortality in patients with cardiovascular disease (14) and malignancies (15). Similarly, in the CKD population, high SII levels independently predict a 15–48% increase in all-cause mortality risk (13). However, SII’s prognostic utility in CKD may be limited: a recent meta-analysis found substantial heterogeneity in the SII-sarcopenia association (I^2^ = 95–96%) and did not include CKD-specific cohorts (16). SII’s components (neutrophil, lymphocyte, and platelet counts) may also be confounded by uremia-related hematologic changes in CKD patients, limiting its specificity as an inflammatory marker in this population—underscoring the need for a tool that directly captures muscle status alongside inflammation. Additionally, C-reactive protein (CRP), a clinically ubiquitous marker of chronic systemic inflammation, is frequently elevated in CKD patients (17, 18). Importantly, elevated CRP is closely linked to the pathogenesis of sarcopenia (19, 20). Given that inflammation is a key driver of muscle catabolism, elevated CRP may reflect not only a toxic systemic environment but also an exacerbation of the “Malnutrition-Inflammation-Cachexia Syndrome” (MICS), thereby synergistically amplifying the adverse prognostic impact of sarcopenia on CKD outcomes (20, 21).

Sarcopenia and systemic inflammation are highly comorbid in CKD, forming a vicious cycle driven by multiple mechanisms, including insulin resistance, mitochondrial dysfunction, accumulation of uremic toxins, and enhanced protein catabolism (22, 23). However, existing literature predominantly assesses these factors in isolation, lacking an integrated tool to quantify their combined effect on prognosis. Therefore, the present study aims to develop a novel SI Index. By integrating functional muscle traits with systemic inflammatory markers (including SII and CRP), this study seeks to provide a more precise risk stratification tool for all-cause mortality in CKD patients and to establish a scientific basis for the early identification of high-risk populations and the formulation of personalized intervention strategies.

## Materials and methods

### Study design and data sources

This study was designed as a prediction model development and validation study: the C-OPTION cohort served as the derivation cohort for constructing and internally validating the SI Index, while the NHANES and CHARLS cohorts served as cross-population generalizability cohorts, in which the pre-specified SI Index formula was applied without re-estimation of its weights. This study employed a prospective cohort design, utilizing data from three distinct databases: the Chinese Observational Prospective Study of Ageing Population with Chronic Kidney Disease (C-OPTION), the National Health and Nutrition Examination Survey (NHANES), and the China Health and Retirement Longitudinal Study (CHARLS).

The C-OPTION study is a multicenter, prospective cohort investigation focusing on the elderly CKD population in China; it served as both the discovery and primary validation dataset for the present analysis. To examine whether the SI Index generalizes across distinct ethnic groups, socioeconomic backgrounds, and healthcare systems, its performance was further assessed in two large population-representative datasets: NHANES, a nationally representative survey conducted by the National Center for Health Statistics in the United States, and CHARLS, a nationally representative longitudinal survey of Chinese adults aged 45 years and older. These analyses were designed as cross-population generalizability assessments rather than formal external validations, evaluating the real-world transportability of the SI Index across diverse global settings. The C-OPTION was approved by the Ethics Committee of the First Medical Center of Chinese PLA General Hospital (approval no. S2016-100-01). Given that both NHANES and CHARLS provide de-identified, publicly available data, this study was exempted from additional institutional ethical review.

### Study population and participant selection

Participants were recruited from the C-OPTION (2016–2024), NHANES (2007–2018), and CHARLS (2011–2020) datasets. Inclusion criteria comprised a clinical diagnosis of chronic kidney disease (CKD) or the satisfaction of at least one of the following laboratory-based definitions: an estimated glomerular filtration rate (eGFR) < 60 mL/min/1.73 m (2) or a urinary albumin-to-creatinine ratio (UACR) > 30 mg/g. The basis for CKD ascertainment differed across cohorts by data availability: in the C-OPTION cohort, CKD status was established through longitudinal clinical diagnosis and medical records; in the NHANES cohort, CKD status was defined using eGFR and UACR from a single study visit; and in the CHARLS cohort, single-measurement eGFR/UACR criteria were supplemented by self-reported, questionnaire-based physician-diagnosed kidney disease.

**Figure 1:**
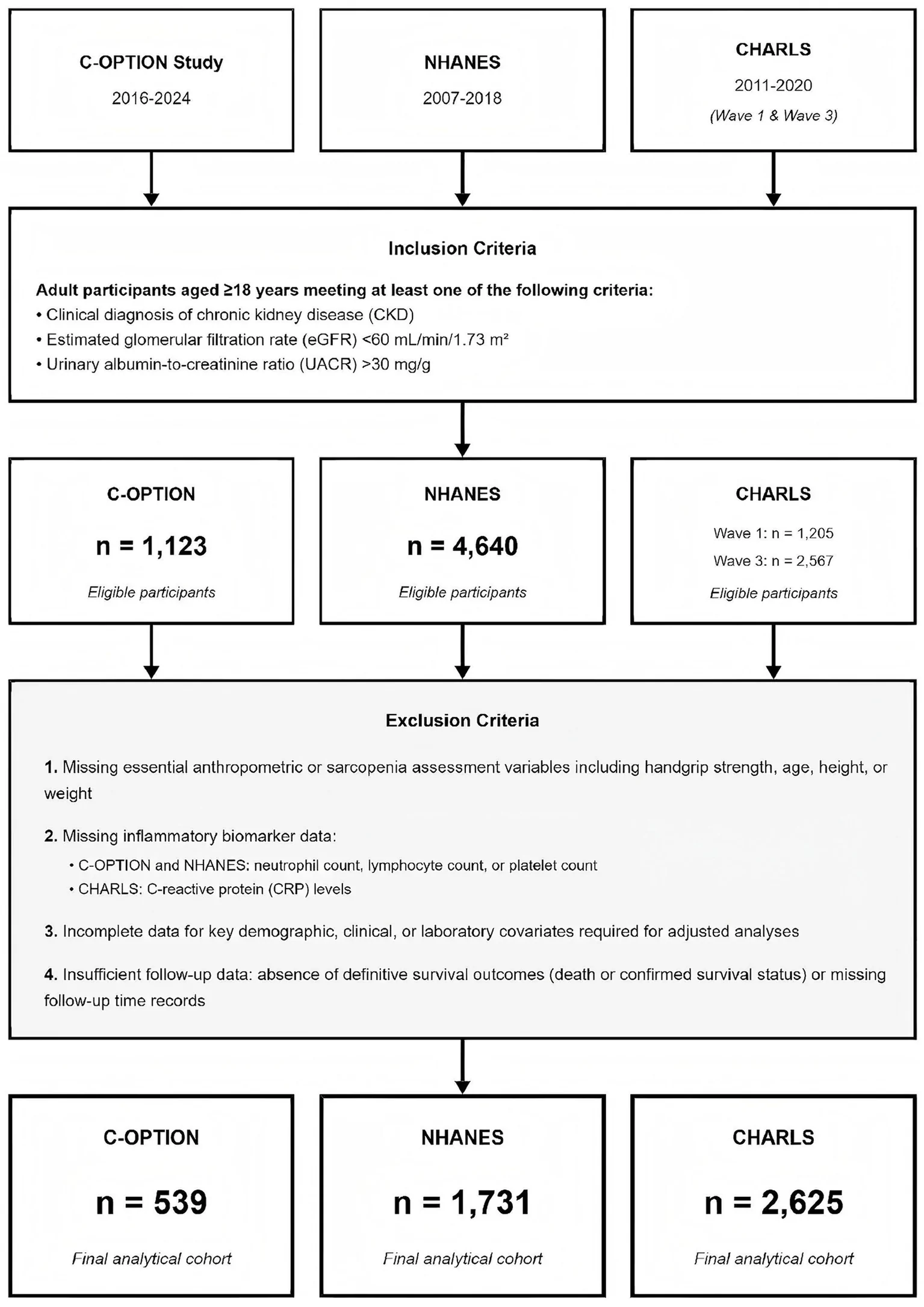
Flow chart of the study participant selection process. C-OPTION, Chinese Observational Prospective Study of Ageing Population with Chronic Kidney Disease; NHANES, National Health and Nutrition Examination Survey; CHARLS, China Health and Retirement Longitudinal Study.

The exclusion criteria were as follows: (1) missing core data required for sarcopenia assessment (grip strength, age, height, and weight) or key variables needed to calculate inflammation indices (neutrophil, lymphocyte, and platelet counts for C-OPTION and NHANES, or CRP levels for CHARLS); (2) unavailable survival outcomes or follow-up duration records; and (3) missing data exceeding 20% across other covariates included in the multivariable models (e.g., biochemical and comorbidity variables). Criteria (1) and (2), pertaining to exposure and outcome ascertainment, were addressed via a complete-case approach, resulting in the exclusion of 584, 2,909, and 1,147 participants in the C-OPTION, NHANES, and CHARLS cohorts, respectively (Figure 1). For participants retained after this step, remaining missing values for the covariates addressed in criterion (3) were handled using multiple imputation by chained equations (MICE), performed separately within each cohort.

Following this screening process, the final study population included 539 participants from the C-OPTION cohort, 1,731 participants from the NHANES cohort, and 2,625 participants from the CHARLS cohort. The detailed participant selection process is illustrated in Figure 1.

### Data collection

This study collected multidimensional data from the baseline survey, including (1) demographic characteristics and basic vital signs, such as age, sex, height, weight, grip strength, body mass index (BMI), systolic blood pressure (SBP), and diastolic blood pressure (DBP); (2) blood biochemical markers; (3) comorbidities; and (4) concomitant medication use.

### Construction of the SI Index

To quantitatively assess the synergistic impact of muscle wasting and chronic inflammation on CKD prognosis, this study developed a novel weighted composite metric—the SI Index. The construction process comprised three steps (Supplementary Figure 1).

### Step 1: initial assessment and classification

#### Sarcopenia assessment

Appendicular skeletal muscle mass (ASM) was estimated using the simplified anthropometric equation developed by Lee et al. (24):

$$\begin{array}{l} \mathrm{ASM}\;(\mathrm{kg})=0.244\times \text{weight}\;(\mathrm{kg})+7.80\times \text{height}\;(m)\\ +6.6\times \mathrm{sex}-0.098\times \mathrm{age}+\text{race}-3.3 \end{array}$$

where sex: male = 1, female = 0; race: Asian = −1.2, Black = 1.4, Hispanic = −0.5, White = 0. Subsequently, the Skeletal Muscle Mass Index (SMI) was calculated as ASM/height^2^ (kg/m^2^) (25).

#### Diagnosis was stratified based on two dimensions

SMI and grip strength. Cohort-specific cut-off points were employed to define low muscle mass and low muscle strength:

#### C-OPTION and CHARLS cohorts

In accordance with Asian Working Group for Sarcopenia (AWGS) 2019 criteria, SMI cut-offs were < 7.0 kg/m^2^ for men and < 5.4 kg/m^2^ for women; grip strength cut-offs were < 28 kg for men and < 18 kg for women (26).

#### NHANES cohort

Based on US population standards, SMI cut-offs were < 7.26 kg/m^2^ for men and < 5.45 kg/m^2^ for women (25); grip strength cut-offs were < 26 kg for men and < 16 kg for women (27).

Participants were categorized into three groups: the Healthy group (normal SMI and grip strength), the Abnormal Muscle Traits (AMT) group (abnormality in either SMI or grip strength), and the Sarcopenia group (abnormalities in both SMI and grip strength).

#### Inflammation assessment

The SII was utilized for the C-OPTION and NHANES cohorts, while CRP was used for the CHARLS cohort. High inflammatory status was defined as SII > 600 (28–30) or CRP > 3 mg/L (31, 32), based on validated criteria from prior cardiovascular studies.

### Step 2: weight determination via cox regression

Multivariate Cox proportional hazards regression models were constructed to determine each risk factor’s relative contribution to mortality; candidate covariates were included after univariate screening (*p* < 0.05) and multicollinearity assessment (VIF < 10). Three key regression coefficients (*β*, log hazard ratio) were extracted: β1 (Abnormal Muscle Traits), β2 (Sarcopenia), and β3 (High Inflammation).

### Step 3: calculation of the weighted index

Using the regression coefficients (β) derived from the discovery cohort, the binary risk variables were weighted and linearly combined to calculate the SI Index:

SIIndex=β1×S1+β2×S2+β3×I

In this index, S1, S2, and I represent the presence (1) or absence (0) of AMT, Sarcopenia, and High Inflammation, respectively; a higher SI Index indicates greater predicted mortality risk. Because the Healthy, AMT, and Sarcopenia groups are mutually exclusive, S1 and S2 cannot both equal 1. The six possible SI Index values (expressed in terms of *β*1, β2, and β3) are presented in Supplementary Table 1; fitted coefficient values are reported in Results (“Construction of the SI Index”).

The SI Index’s β coefficients were derived exclusively from the C-OPTION cohort and subsequently applied uniformly across all validation cohorts to assess external generalizability.

The primary outcome was all-cause mortality during the follow-up.

### Statistical analysis

Continuous variables were assessed for normality using the Shapiro–Wilk test. Skewed data were expressed as medians with interquartile ranges (IQR) and compared between groups using the Mann–Whitney U test. Categorical variables were presented as frequencies (percentages), with between-group comparisons performed using the Chi-square test or Fisher’s exact test when expected frequencies were below five. Kaplan–Meier (K-M) curves and Log-rank tests were employed to evaluate cumulative survival rates across strata of sarcopenia status and inflammation levels. Univariate and multivariate Cox proportional hazards regression models were constructed to identify independent risk factors for all-cause mortality. Multicollinearity was assessed using the Variance Inflation Factor (VIF), with a threshold of < 10. The three cohorts were not matched or harmonized; between-cohort baseline differences were addressed analytically, with variables that were significant (*p* < 0.05) and free of collinearity in each cohort’s univariate analysis entered as covariates into that cohort’s multivariable model. Results are reported as hazard ratios (HR) with 95% confidence intervals (CI).

The SI Index was established based on regression coefficients (*β*) derived from the multivariate Cox model within the C-OPTION (discovery) cohort. The index was designed as a weighted linear combination of independent risk factors. To evaluate external generalizability, this formula was subsequently applied to the NHANES and CHARLS validation cohorts.

Predictive performance and discrimination were evaluated using time-dependent Receiver Operating Characteristic (ROC) curves—calculating the area under the curve (AUC) at 1, 3, 5, 7, and 9 years—and the Concordance Index (C-index). Model goodness-of-fit and complexity were assessed via the Akaike Information Criterion (AIC) and Bayesian Information Criterion (BIC). The incremental prognostic value of the SI Index was quantified by comparing metrics (AUC, C-index, AIC, and BIC) between a base model (adjusted for cohort-specific clinical covariates) and a combined model (base model + SI Index). Furthermore, 10-fold cross-validation was conducted within each cohort to verify C-index stability and minimize overfitting. Notably, the 10-fold cross-validation performed within the C-OPTION cohort served as the internal validation of the SI Index, confirming model stability and generalizability prior to cross-population generalizability assessment in the NHANES and CHARLS datasets. Subgroup analyses and interaction tests were performed to assess the consistency of the SI Index’s predictive efficacy across diverse clinical strata.

Model calibration was assessed using calibration plots at multiple time points. Analysis was stratified by six discrete levels of the SI Index to compare risks predicted by the Cox model against observed risks estimated via the Kaplan–Meier method. Calibration performance was quantified using the calibration slope (ideal value: 1.0), calibration intercept (ideal value: 0), and R^2^.

All statistical analyses were performed using R software (version 4.5.1, The R Foundation for Statistical Computing). Statistical significance was defined as a two-sided *p* < 0.05. Key R packages utilized included tableone, survival, survminer, timeROC, rms, Hmisc, gridExtra, openxlsx, forestplot, and ggplot2.

## Results

### Baseline characteristics of the study cohorts

A comprehensive comparison of baseline characteristics across all three cohorts is provided in Supplementary Table 4. The baseline characteristics of the primary discovery cohort (C-OPTION, *N* = 539) and the two cross-population generalizability cohorts (NHANES, *N* = 1,731; CHARLS, *N* = 2,625) are detailed in Table 1, Supplementary Tables S2, S3, respectively. All data are stratified by survival status.

**Table 1:** Baseline characteristics of patients with chronic kidney disease (CKD) in the C-OPTION Cohort, stratified by survival status.

Items	Survive (*N* = 494)	Death (*N* = 45)	Total (*N* = 539)	*p*-value
Age (years)	67.00 (64.00, 73.00)	68.00 (65.00, 75.00)	68.00 (64.00, 74.00)	0.119
Sex (male)	308 (62.9%)	34 (77.3%)	342 (64.0%)	0.081
Height (cm)	165.00 (159.00, 171.20)	165.00 (160.00, 171.00)	165.00 (159.00, 171.00)	0.479
Weight (kg)	68.00 (60.20, 75.50)	67.70 (63.00, 77.50)	68.00 (61.00, 75.90)	0.486
BMI	24.80 (22.64, 27.31)	24.87 (23.29, 27.85)	24.80 (22.66, 27.34)	0.555
SBP (mmHg)	137.00 (125.00, 147.50)	145.00 (127.00, 158.50)	137.00 (125.25, 148.50)	0.106
DBP (mmHg)	79.00 (73.50, 87.50)	81.50 (74.00, 93.50)	79.50 (73.50, 87.50)	0.229
Grip-max (kg)	27.40 (21.00, 36.10)	22.40 (17.30, 28.70)	27.00 (20.60, 35.80)	0.001
Low-grip (*n*, %)	150 (30.4%)	28 (62.2%)	178 (33.0%)	<0.001
SMI (kg/m^2^)	7.40 (6.38, 8.05)	7.67 (7.02, 8.07)	7.42 (6.40, 8.05)	0.172
Low-SMI (*n*, %)	33 (6.7%)	3 (6.7%)	36 (6.7%)	0.997
Laboratory examination				
HB (g/L)	120.50 (106.00, 134.00)	115.50 (103.00, 125.00)	120.00 (105.50, 133.00)	0.046
WBC (10^9/L)	6.39 (5.30, 7.83)	7.32 (5.81, 8.30)	6.45 (5.33, 7.90)	0.128
Neutrophils (%)	61.90 (55.35, 69.00)	63.90 (55.35, 70.68)	62.00 (55.35, 69.30)	0.277
Lymphocytes (%)	27.00 (21.00, 33.60)	24.15 (19.55, 31.25)	26.80 (20.80, 33.40)	0.132
PLT (10^9/L)	204.00 (168.00, 251.00)	204.00 (150.00, 239.00)	204.00 (167.00, 248.00)	0.356
TC (mmol/L)	4.84 (3.93, 6.14)	4.82 (3.98, 6.14)	4.83 (3.94, 6.14)	0.719
TG (mmol/L)	1.69 (1.17, 2.45)	1.59 (0.96, 2.49)	1.68 (1.17, 2.45)	0.616
HDL (mmol/L)	1.15 (0.98, 1.43)	1.16 (0.92, 1.33)	1.15 (0.97, 1.42)	0.427
LDL (mmol/L)	2.91 (2.21, 3.86)	2.87 (2.18, 4.02)	2.91 (2.21, 3.87)	0.761
GLU (mmol/L)	5.13 (4.57, 6.12)	4.86 (4.37, 6.44)	5.13 (4.54, 6.12)	0.392
BUN (mmol/L)	8.52 (6.40, 12.30)	9.22 (7.22, 13.35)	8.60 (6.41, 12.51)	0.373
UA (umol/L)	399.00 (333.00, 470.00)	385.00 (335.70, 458.40)	398.00 (333.40, 470.00)	0.985
SCR (umol/L)	121.00 (81.10, 180.00)	135.45 (79.55, 203.45)	121.50 (81.00, 180.15)	0.512
eGFR (mL/min/1.73 m^2^)	48.84 (27.54, 76.56)	43.78 (25.81, 82.41)	48.81 (27.38, 76.94)	0.595
CKD stage (*n*, %)				0.967
G1 (≥90 mL/min/1.73 m^2^)	81 (16.6%)	6 (14.0%)	87 (16.4%)	
G2 (60–89)	97 (19.9%)	9 (20.9%)	106 (20.0%)	
G3a (45–59)	86 (17.6%)	6 (14.0%)	92 (17.3%)	
G3b (30–44)	87 (17.8%)	8 (18.6%)	95 (17.9%)	
G4 (15–29)	112 (23.0%)	12 (27.9%)	124 (23.4%)	
G5 (<15)	25 (5.1%)	2 (4.7%)	27 (5.1%)	
UTP (g/24 h)	1.96 (0.55, 4.72)	3.42 (1.32, 5.04)	2.08 (0.60, 4.79)	0.034
SII	461.12 (311.63, 716.45)	605.95 (387.41, 910.57)	471.61 (313.55, 724.91)	0.032
High Inflammation (*n*, %)	159 (32.2%)	23 (51.1%)	182 (33.8%)	0.016
Comorbidities				
Hypertension (*n*, %)	381 (77.1%)	39 (86.7%)	420 (77.9%)	0.197
Diabetes (*n*, %)	175 (35.4%)	16 (35.6%)	191 (35.4%)	>0.999
Heart disease (*n*, %)	86 (17.4%)	11 (24.4%)	97 (18.0%)	0.330
Stroke (*n*, %)	85 (17.2%)	16 (35.6%)	101 (18.7%)	0.005
Medicine				
Antihypertensive Drug (*n*, %)	397 (80.4%)	38 (84.4%)	435 (80.7%)	0.641
Antidiabetic drug (*n*, %)	151 (30.6%)	16 (35.6%)	167 (31.0%)	0.600
Lipid-lowering drug (*n*, %)	198 (40.1%)	21 (46.7%)	219 (40.6%)	0.482
Smoking (*n*, %)	115 (23.3%)	15 (33.3%)	130 (24.1%)	0.143
Drinking (*n*, %)	98 (19.8%)	10 (22.2%)	108 (20.0%)	0.728
Sarcopenia status (*n*, %)				<0.001
Healthy (*n*, %)	340 (68.8%)	17 (37.8%)	357 (66.2%)	
Abnormal muscle traits (*n*, %)	125 (25.3%)	25 (55.6%)	150 (27.8%)	
Sarcopenia (*n*, %)	29 (5.9%)	3 (6.7%)	32 (5.9%)	
Follow-time (years)	1.63 (0.91, 3.20)	1.38 (0.60, 4.70)	1.59 (0.88, 3.25)	0.379

BMI: Body Mass Index; BUN: Blood Urea Nitrogen; CKD: Chronic Kidney Disease; C-OPTION: Chinese Observational Prospective Study of Ageing Population with Chronic Kidney Disease; DBP: Diastolic Blood Pressure; eGFR: Estimated Glomerular Filtration Rate; GLU: Glucose; HB: Hemoglobin; HDL: High-Density Lipoprotein Cholesterol; LDL: Low-Density Lipoprotein Cholesterol; PLT: Platelet Count; SBP: Systolic Blood Pressure; SCR: Serum Creatinine; SII: Systemic Immune-Inflammation Index; SMI: Skeletal Muscle Mass Index; TC: Total Cholesterol; TG: Triglycerides; UA: Uric Acid; UTP: 24-h Urinary Total Protein; WBC: White Blood Cell Count.

Primary Discovery Cohort In the C-OPTION cohort (median follow-up: 1.59 years), 45 deaths (8.3%) were recorded. The prevalence of sarcopenia, AMT, and high inflammation was 5.9% (32/539), 27.8% (150/539), and 33.8% (182/539), respectively. Compared to survivors, non-survivors exhibited significantly impaired muscle function (maximum grip strength: 22.40 vs. 27.40 kg, *p* = 0.001; low-grip-strength incidence: 62.2% vs. 30.4%, *p* < 0.001), higher inflammatory levels (elevated SII, *p* = 0.032; high inflammation proportion: 51.1% vs. 32.2%, *p* = 0.016), and a significantly different distribution of sarcopenia status (*p* < 0.001; AMT proportion in non-survivors: 55.6% vs. 25.3%). Additionally, non-survivors had lower hemoglobin levels, higher urine total protein (UTP), and a higher prevalence of stroke (all *p* < 0.05).

Validation Cohorts in the NHANES cohort (median follow-up: 6.42 years), 460 deaths (26.6%) occurred. The prevalence of sarcopenia, AMT, and high inflammation was 2.5% (44/1,731), 13.8% (150/1,731), and 37.8% (654/1,731), respectively. Non-survivors showed significantly poorer muscle function (SMI, grip strength) and higher inflammatory levels (SII) (all *p* < 0.001), along with older age and a higher proportion of males (*p* = 0.007). Renal function and metabolic profiles were generally worse among non-survivors (e.g., elevated glucose, uric acid, and creatinine, with reduced eGFR), and the prevalence of comorbidities (hypertension, diabetes, heart disease, stroke) and smoking was also significantly higher (all *p* < 0.001).

In the CHARLS cohort (median follow-up: 5.00 years), 367 deaths (14.0%) were recorded. The prevalence of sarcopenia, AMT, and high inflammation was 3.4% (88/2,625), 24.3% (638/2,625), and 22.8% (599/2,625), respectively. Non-survivors showed significantly poorer muscle function and inflammatory levels (CRP) (all *p* < 0.01–0.001), along with older age and a higher proportion of males (*p* < 0.001). As in NHANES, metabolic and renal profiles were generally worse among non-survivors, and the prevalence of comorbidities as well as smoking and alcohol consumption rates were also significantly higher (all *p* < 0.05).

In addition, a comprehensive comparison of baseline characteristics across all three cohorts is provided in Supplementary Table 4. Baseline comorbidity burden and medication use differed substantially across cohorts (all *p* < 0.001).

#### Kaplan–Meier survival analysis

**Figure 2:**
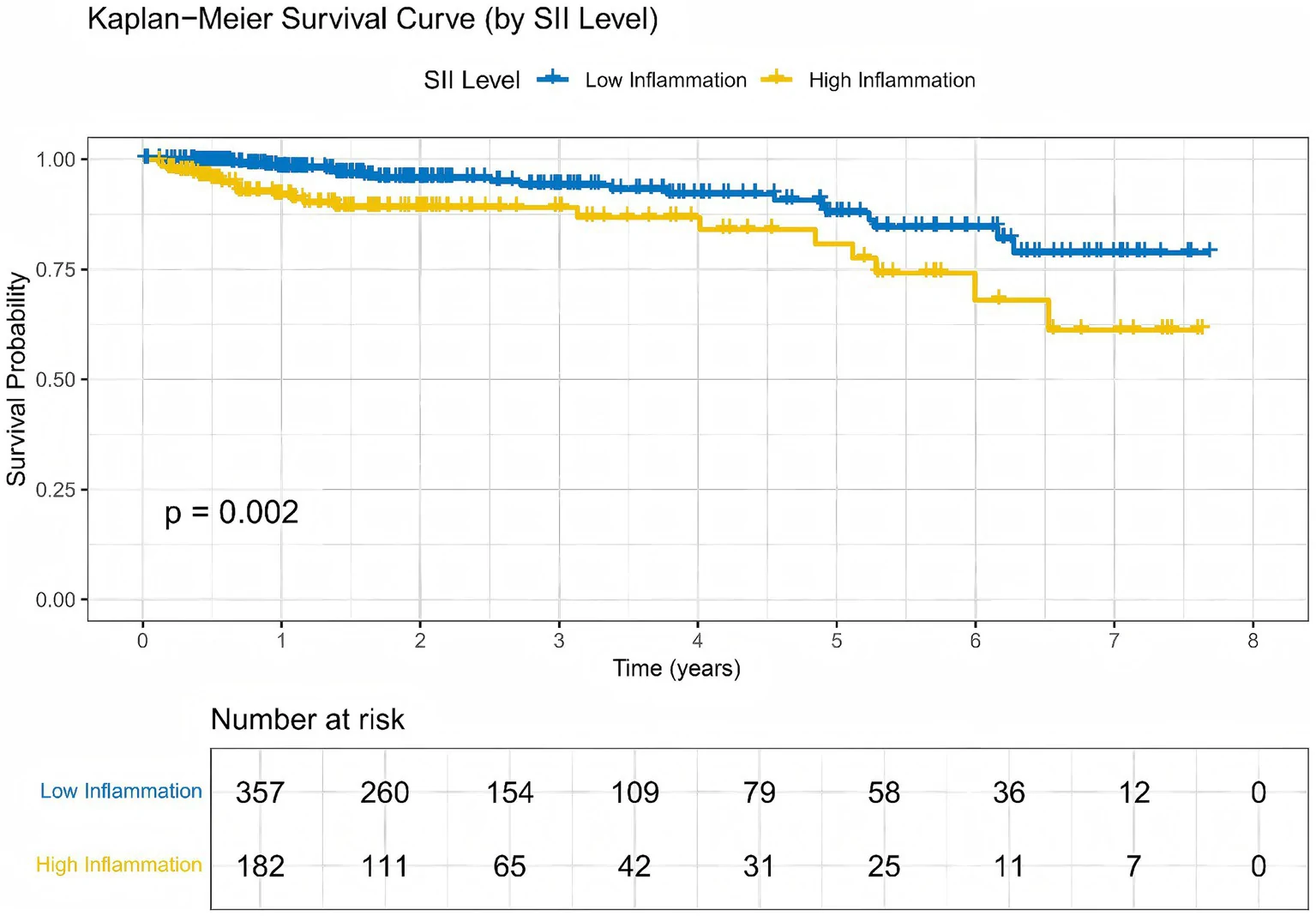
Kaplan–Meier survival curves for all-cause mortality stratified by SII levels in the C-OPTION cohort. SII, Systemic Immune-Inflammation Index.

**Figure 3:**
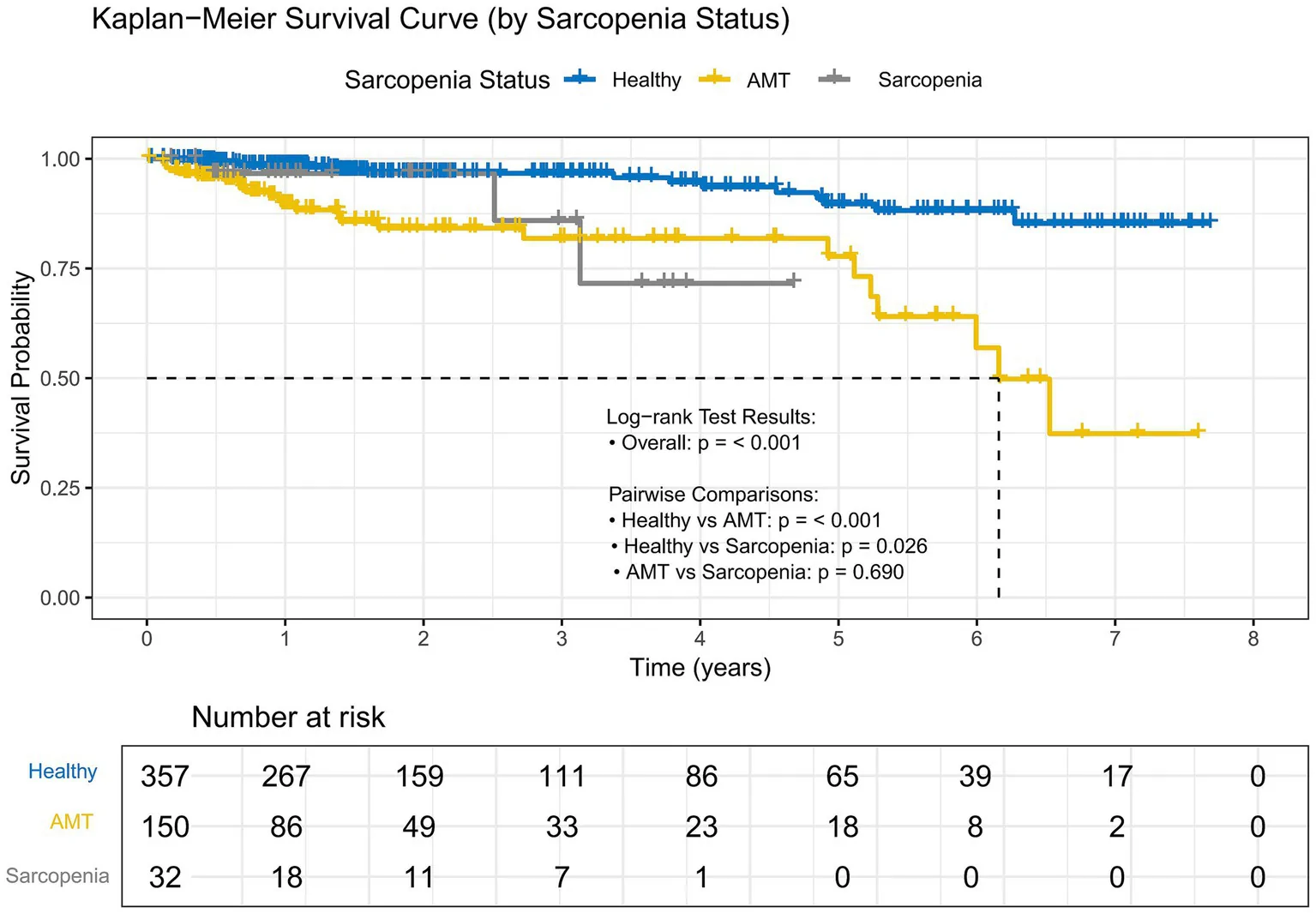
Kaplan–Meier survival analysis according to sarcopenia status in the C-OPTION cohort. AMT, Abnormal Muscle Traits.

Kaplan–Meier survival analysis showed that in the C-OPTION cohort, the cumulative survival rate in the high-inflammation group was significantly lower than in the low-inflammation group (Log-rank *p* = 0.002; Figure 2); survival differed significantly across the three muscle status groups (Log-rank *p* < 0.001), with both the Sarcopenia and AMT groups showing significantly lower survival than the Healthy group (both *p* < 0.05), though the difference between the Sarcopenia and AMT groups did not reach statistical significance (*p* = 0.690; Figure 3).

Results were consistent in the NHANES and CHARLS cohorts, where both high inflammation and sarcopenia were significantly associated with all-cause mortality (Log-rank *p* < 0.001; Supplementary Figures 2–5); unlike C-OPTION, the Sarcopenia group showed significantly lower survival than the AMT group in these two cohorts (pairwise comparisons *p* < 0.05).

#### Univariate cox regression analysis

**Figure 4:**
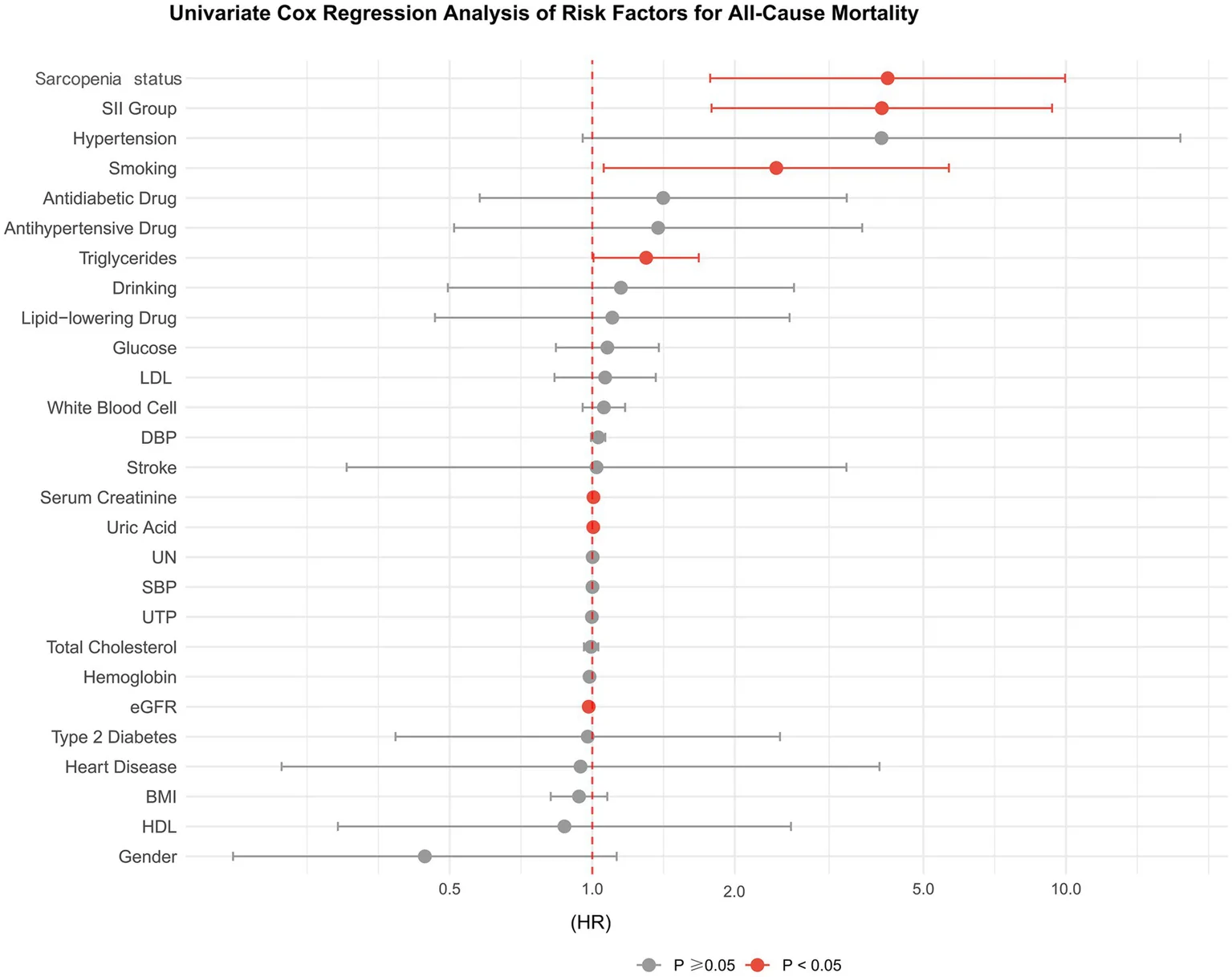
Univariate cox regression analysis of risk factors for all-cause mortality in the C-OPTION cohort. BMI, Body Mass Index; C-OPTION, Chinese Observational Prospective Study Of Ageing Population With Chronic Kidney Disease; CI, Confidence Interval; DBP, Diastolic Blood Pressure; eGFR, Estimated Glomerular Filtration Rate; HDL, High-Density Lipoprotein; HR, Hazard Ratio; LDL, Low-Density Lipoprotein; SBP, Systolic Blood Pressure; SII, Systemic Immune-Inflammation Index; UN, Urea Nitrogen; UTP, Urinary Total Protein.

Univariate Cox regression analyses consistently showed that both sarcopenia and inflammatory markers (SII/CRP) were significantly and positively associated with mortality risk (HR > 1.0) across the C-OPTION, NHANES, and CHARLS cohorts, with their prognostic impact generally exceeding that of traditional clinical risk factors—such as hypertension, diabetes, and heart disease—and renal biochemical markers such as serum creatinine and uric acid (Figure 4; Supplementary Figures 6, 7).

#### Multivariate cox regression analysis and construction of the SI Index

After excluding indicators exhibiting multicollinearity (VIF > 10: BUN, SCR, and TC in NHANES; SBP, BUN, and TC in CHARLS) and incorporating all variables with *p* < 0.05 from the univariate analysis, multivariate Cox regression analyses were performed separately across the three cohorts (Table 2; Supplementary Tables S5, S6). In the C-OPTION cohort, Model 3 revealed no significant interaction between sarcopenia and high inflammation; consequently, the main effects model (Model 2) was adopted.

**Table 2:** Multivariate Cox regression analysis of sarcopenia status, inflammation, and associated factors with all-cause mortality in the C-OPTION Cohort.

Factors	Model 1			Model 2				Model 3		
	HR	95%CI	*p*-value	HR	95%CI	*p*-value	β	HR	95%CI	*p*-value
Sarcopenia (*n*,%)	3.561	1.023–12.396	0.046	4.582	1.178–17.826	0.028	1.522	7.034	1.364–36.267	0.020
Abnormal Muscle Traits (*n*,%)	4.087	2.174–7.686	<0.001	3.671	1.553–8.675	0.003	1.300	2.997	0.845–10.635	0.089
High inflammation (*n*,%)	1.824	1.002–3.321	0.049	2.411	1.052–5.526	0.037	0.880	3.036	0.923–9.981	0.068
UA (umol/L)				1.004	1.001–1.007	0.022				
SCR (umol/L)				1.002	0.997–1.007	0.479				
Sarcopenia × High inflammation								1.077	0.194–5.979	0.932
Abnormal muscle traits× High inflammation								0.281	0.019–4.115	0.354

Model 1 (crude) includes Sarcopenia, Abnormal Muscle Traits, and High Inflammation only. Model 2 (adjusted) additionally includes UA and SCR. Model 3 additionally includes the two-way interaction terms between muscle status (Sarcopenia, Abnormal Muscle Traits) and High Inflammation. HR: Hazard Ratio, 95% CI: 95% Confidence Interval, *p*-value: Probability value, β: Beta coefficient (Log Hazard Ratio, ln{HR}), UA: Uric Acid, SCR: Serum Creatinine.

Sarcopenia was the strongest predictor of all-cause mortality (HR 4.582–5.947); AMT independently and significantly increased mortality risk across all three cohorts (HR 2.464–3.671, all *p* ≤ 0.003); high inflammatory status was likewise an independent risk factor across all three cohorts (HR 1.425–2.411, all *p* ≤ 0.037). The risk effects of sarcopenia and inflammation consistently exceeded those of other clinical and biochemical indicators (Table 2; Supplementary Tables S5, S6).

#### Construction of the SI Index

To quantify the combined risk contribution of sarcopenia status and high inflammation to all-cause mortality, this study constructed the SI Index based on the C-OPTION cohort. The index employed the regression coefficients *β* (i.e., log hazard ratios) of each independent risk factor from the multivariate Cox model (Model 1) as weights for a linear combination. The specific formula established is as follows:

$$\begin{array}{l} \mathrm{SI}\;\text{Index}=1.522\times (\text{Sarcopenia})+1.300\\ \times (\text{Abnormal Muscle Traits})+0.880\times (\text{High Inflammation}) \end{array}$$

This formula was subsequently applied to calculate the SI Index for participants in the NHANES and CHARLS validation cohorts.

#### Validation of predictive performance and discrimination of the SI Index across multiple cohorts

**Figure 5:**
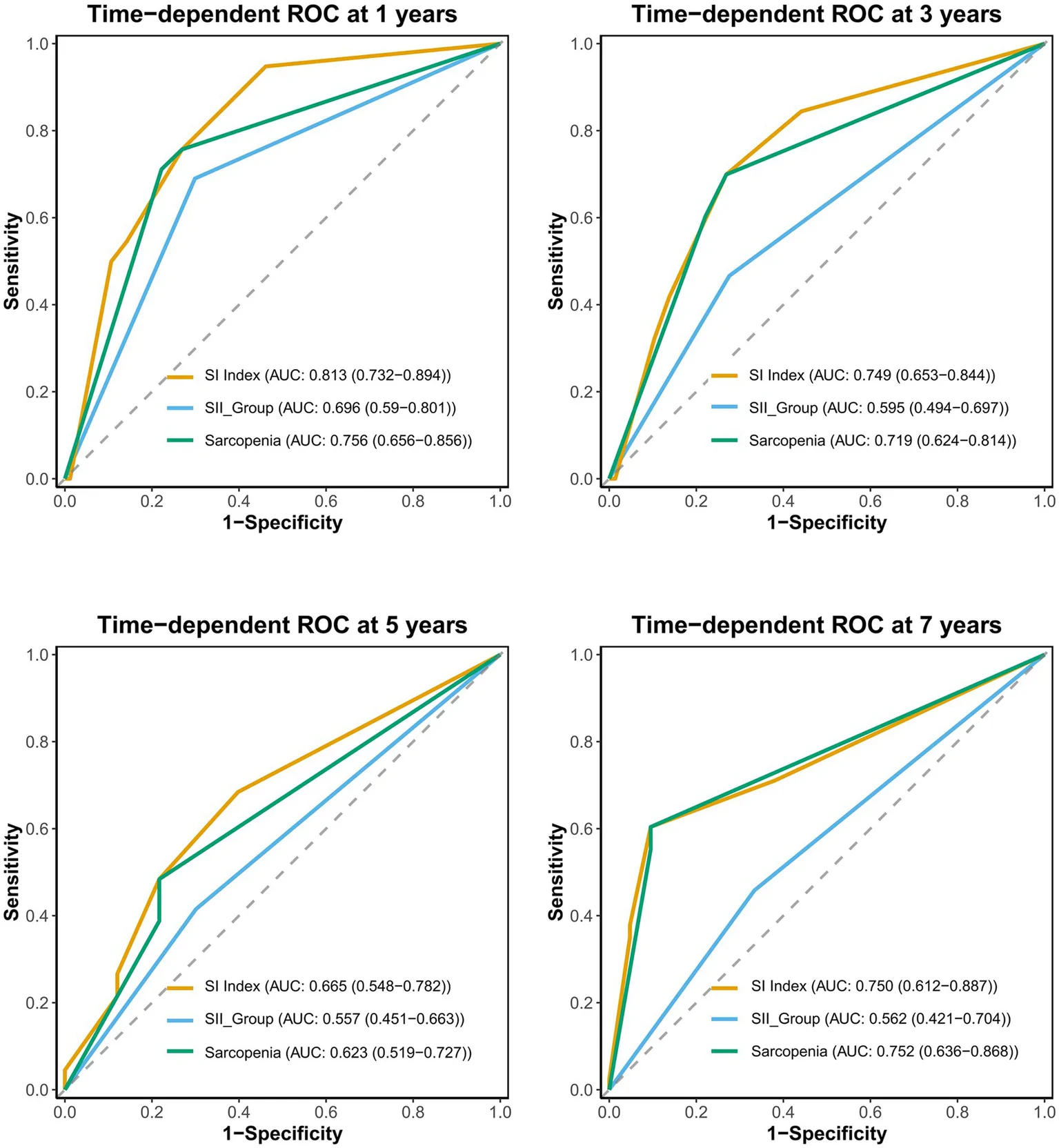
Time-dependent ROC curves illustrating the predictive performance of the SI Index, sarcopenia status, and SII Group for mortality in the C-OPTION cohort at 1, 3, 5, and 7 years. SII, Systemic Immune-Inflammation Index; SI Index, Sarcopenia-Inflammation Index.

Time-dependent ROC analysis showed that the SI Index consistently outperformed models based on sarcopenia or inflammatory markers alone (Figure 5). In the discovery cohort, the SI Index achieved the highest AUC throughout follow-up, peaking at year 1 (AUC = 0.813) and remaining robust at year 7 (AUC = 0.750), compared to limited predictive value for the sarcopenia and SII groups alone (Table 3). Given this cohort’s short median follow-up (1.59 years) and markedly reduced risk set at later time points (only 19 of 539 patients remained at risk at 7 years; Supplementary Table 7), the 5- and 7-year estimates should be interpreted as evidence of consistent discriminative trends rather than precise long-term risk estimates.

**Table 3:** Comparison of predictive accuracy and model fit for the SI Index versus sarcopenia and inflammatory markers across the C-OPTION, NHANES, and CHARLS cohorts.

Factors		AUC					AIC	BIC
		1 year	3 year	5 year	7 year	9 year		
C-OPTION	SI Index	0.813 (0.732–0.894)	0.749 (0.653–0.844)	0.665 (0.548–0.782)	0.750 (0.612–0.887)		457.6	459.41
	Sarcopenia status	0.756 (0.656–0.856)	0.719 (0.624–0.814)	0.623 (0.519–0.727)	0.752 (0.636–0.868)		462.78	466.39
	SII_Group	0.696 (0.590–0.801)	0.595 (0.494–0.697)	0.557 (0.451–0.663)	0.562 (0.421–0.704)		477.47	479.27
NHANES	SI Index	0.755 (0.692–0.819)	0.699 (0.659–0.740)	0.683 (0.650–0.716)	0.679 (0.648–0.711)		6421.49	6442.15
	Sarcopenia status	0.696 (0.629–0.763)	0.664 (0.627–0.701)	0.652 (0.624–0.680)	0.645 (0.620–0.671)		6428.40	6436.66
	SII_Group	0.600 (0.534–0.666)	0.566 (0.529–0.603)	0.556 (0.526–0.586)	0.563 (0.532–0.593)		6590.96	6595.09
CHARLS	SI Index		0.693 (0.655–0.732)	0.711 (0.676–0.746)	0.710 (0.677–0.743)	0.689 (0.654–0.724)	5375.03	5378.9
	Sarcopenia status		0.652 (0.614–0.690)	0.673 (0.639–0.706)	0.676 (0.645–0.707)	0.663 (0.631–0.695)	5393.77	5401.54
	CRP_Group		0.582 (0.546–0.618)	0.584 (0.551–0.616)	0.574 (0.545–0.604)	0.563 (0.533–0.592)	5496.54	5500.43

SI Index, Sarcopenia-Inflammation Index; SII, Systemic Immune-Inflammation Index; CRP, C-Reactive Protein; AUC, Area Under The Curve; AIC, Akaike Information Criterion; BIC, Bayesian Information Criterion; C-OPTION, Chinese Observational Prospective Study of Ageing Population with Chronic Kidney Disease; NHANES, National Health and Nutrition Examination Survey; CHARLS, China Health and Retirement Longitudinal Study.

**Figure 6:**
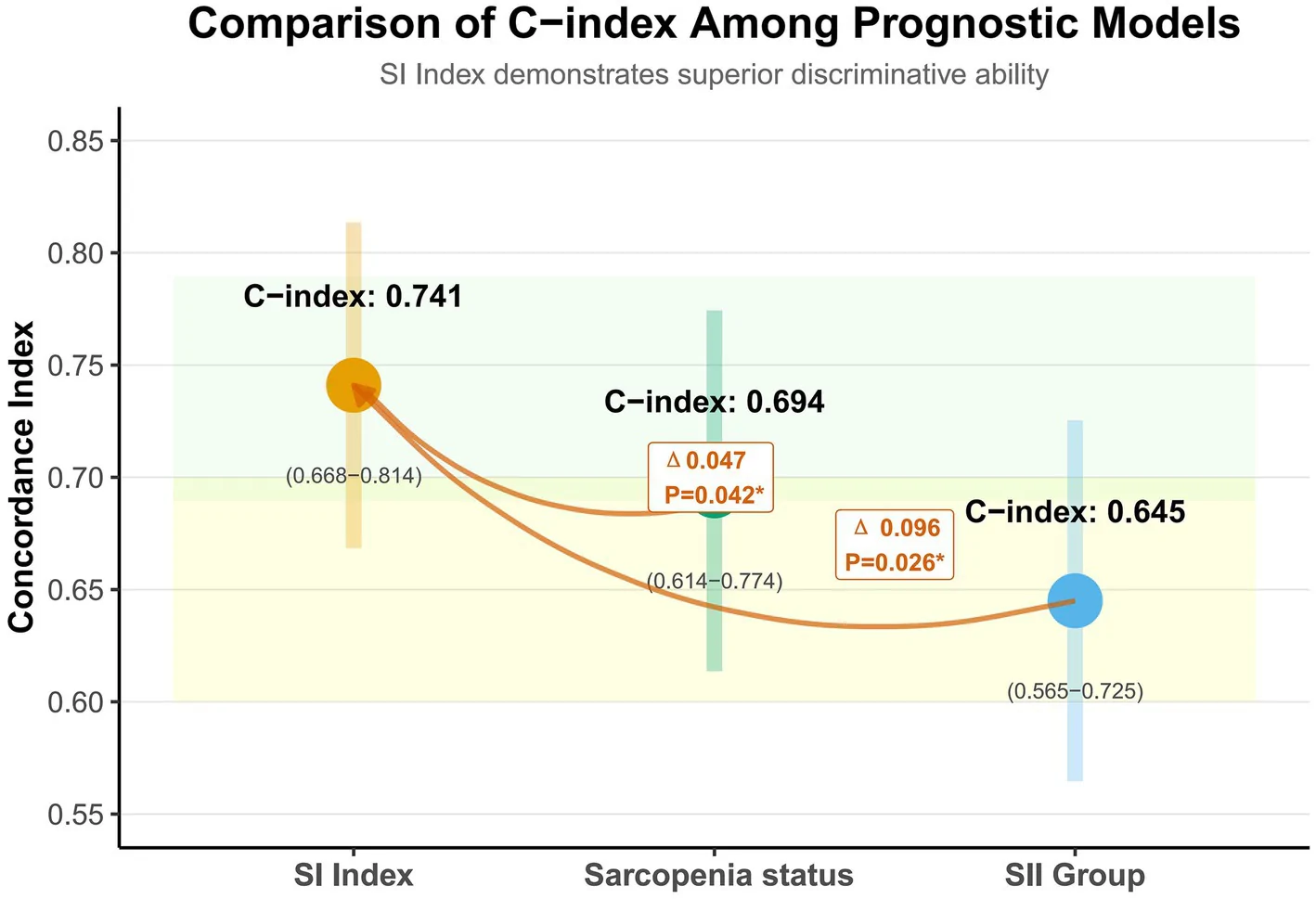
Comparison of the concordance index (C-index) for the SI Index, sarcopenia status, and SII group in the C-OPTION cohort. C-OPTION, Chinese Observational Prospective Study of Ageing Population with Chronic Kidney Disease; SII, Systemic Immune-Inflammation Index; SI Index, Sarcopenia-Inflammation Index.

This advantage was confirmed externally: in NHANES, the SI Index’s AUC (0.679–0.755) consistently exceeded that of sarcopenia (0.645–0.696) and SII (0.556–0.600) alone (Supplementary Figure 8); in CHARLS, the SI Index similarly outperformed sarcopenia and CRP alone across years 3–9 (Supplementary Figure 9). The C-index confirmed superior overall discrimination for the SI Index across all cohorts (0.657–0.741), significantly higher than single-indicator models (all *p* < 0.05) (Figure 6; Supplementary Figures 10, 11). The SI Index also achieved the lowest AIC/BIC across cohorts, indicating an optimal balance between model fit and complexity without overfitting.

#### Incremental predictive value of the SI Index over base clinical models

Predictive performance of the base model versus the combined model (base model + SI Index) is presented in Table 4.

**Table 4:** Comparison of predictive performance and model fit between the base model and the combined model incorporating the SI Index across the C-OPTION, NHANES, and CHARLS cohorts.

Factors		AUC					C-index	P for C-index	AIC	BIC
		1 year	3 year	5 year	7 year	9 year				
C-OPTION	Base model	0.551 (0.408–0.694)	0.633 (0.516–0.750)	0.693 (0.579–0.808)	0.687 (0.530–0.844)		0.565 (0.466–0.663)	<0.001	470.60	474.17
	Combined model	0.784 (0.678–0.890)	0.755 (0.649–0.861)	0.705 (0.591–0.819)	0.777 (0.644–0.910)		0.729 (0.644–0.814)		448.16	453.51
NHANES	Base model	0.736 (0.637–0.835)	0.706 (0.643–0.769)	0.706 (0.659–0.754)	0.712 (0.662–0.761)		0.686 (0.649–0.724)	0.002	2476.69	2489.92
	Combined model	0.861 (0.806–0.916)	0.777 (0.720–0.834)	0.741 (0.693–0.789)	0.774 (0.729–0.819)		0.729 (0.693–0.766)		2414.35	2430.89
CHARLS	Base model		0.734 (0.699–0.768)	0.715 (0.680–0.749)	0.718 (0.686–0.750)	0.733 (0.701–0.764)	0.726 (0.700–0.751)	<0.001	5403.79	5427.22
	Combined model		0.785 (0.751–0.818)	0.783 (0.751–0.815)	0.783 (0.753–0.813)	0.787 (0.758–0.816)	0.768 (0.743–0.794)		5290.02	5317.36

The Base models were adjusted for cohort-specific covariates as follows: C-OPTION, UA and SCR; NHANES, History Of Stroke And Heart Disease, GLU, DBP, UACR, and eGFR; CHARLS, DBP, WBC, GLU, BMI, eGFR, and Gender. The Combined model included the Base model variables plus the SI Index. UA, Uric Acid; SCR, Serum Creatinine; GLU, Blood Glucose; DBP, Diastolic Blood Pressure; UACR, Urine Albumin-To-Creatinine Ratio; eGFR, estimated Glomerular Filtration Rate; WBC, White Blood Cell Count; BMI, Body Mass Index; AUC, Area Under The Curve; AIC, Akaike Information Criterion; BIC, Bayesian Information Criterion.

**Figure 7:**
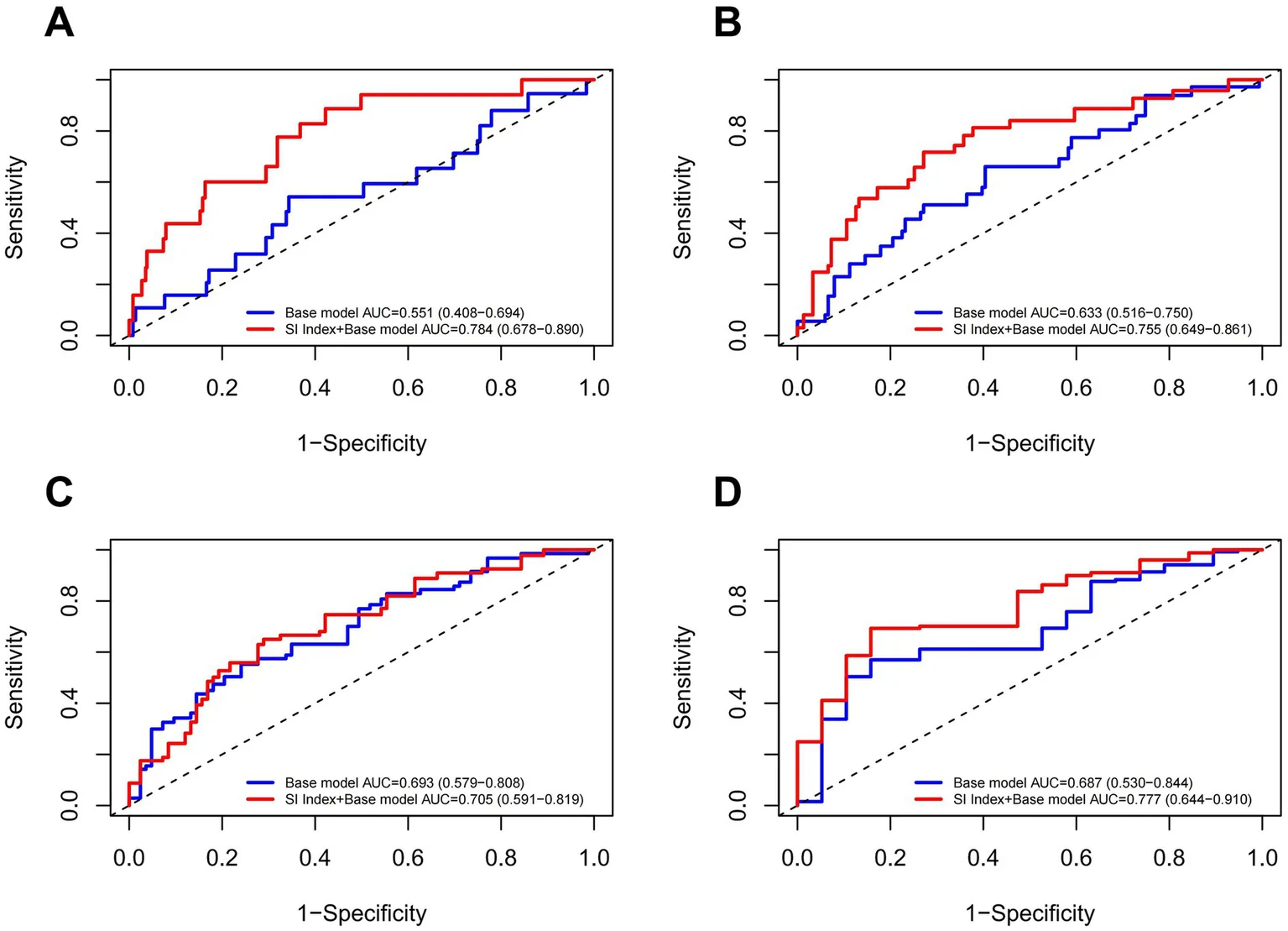
Time-dependent ROC analysis comparing the predictive performance of the Base model and the Combined model for all-cause mortality in the C-OPTION cohort. **(A)** 1-year, **(B)** 3-year, **(C)** 5-year, and **(D)** 7-year ROC curves. The blue line represents the Base model, and the red line represents the Combined model. The base models were adjusted for UA and SCR; The combined model was Base model incorporating the SI Index. C-OPTION, Chinese Observational Prospective Study of Ageing Population with Chronic Kidney Disease; SI Index, Sarcopenia-Inflammation Index.

C-OPTION cohort (Figure 7): base model AUC = 0.551 at year 1, combined model AUC = 0.784; base model AUC = 0.687 at year 7, combined model AUC = 0.777. In the NHANES (Supplementary Figure 12) and CHARLS (Supplementary Figure 13) cohorts, the combined model’s AUC exceeded that of the base model at all time points. The combined model’s C-index was significantly higher than the base model’s (all *p* < 0.001), with lower AIC and BIC (Table 4).

10-fold cross-validation (Supplementary Figures 14–16): within the C-OPTION cohort, the combined model’s C-index was consistently higher than the base model’s; the combined model outperformed the base model across all three cohorts.

Calibration analysis: slope 0.714–0.756 in the C-OPTION cohort (Supplementary Figure 17); slope 1.044–1.395, R^2^ 0.416–0.885 in NHANES (Supplementary Figure 18); slope 1.020–1.264, R^2^ 0.899–0.967 in CHARLS (Supplementary Figure 19).

#### Subgroup analysis

**Figure 8:**
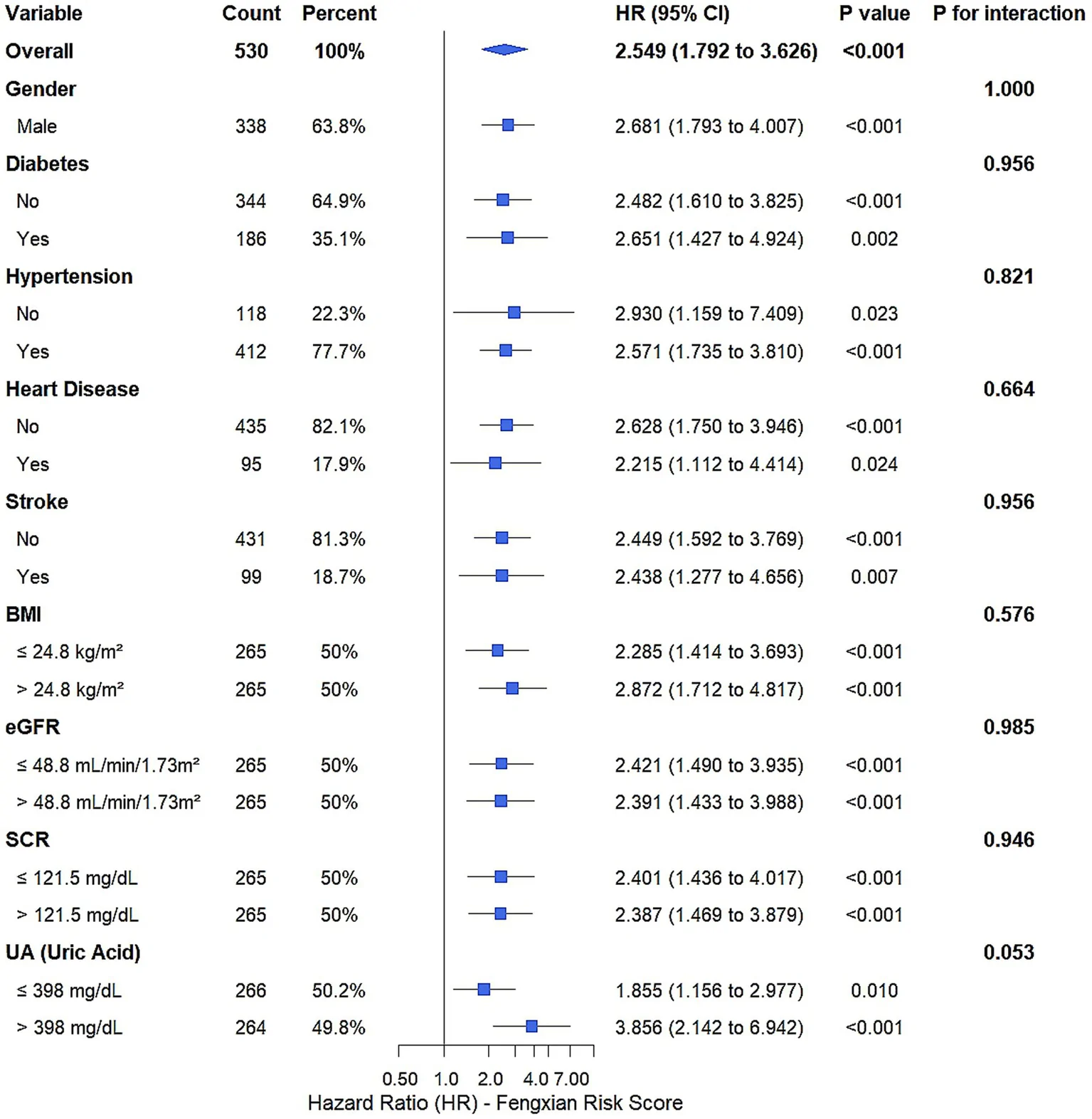
Subgroup analysis of the association between the SI Index and all-cause mortality in the C-OPTION cohort. C-OPTION, Chinese Observational Prospective Study of Ageing Population with Chronic Kidney Disease.

Stratified subgroup analyses were conducted based on sex, comorbidities (diabetes, hypertension, heart disease, and stroke), and the median values of BMI, eGFR, SCR, and UA (Figure 8). The results demonstrated that the significant positive association between the SI Index and all-cause mortality remained robust across all prespecified subgroups. Within each stratum, an elevated SI Index was associated with a significantly increased risk of mortality (all *p* < 0.05). Furthermore, interaction analyses indicated that the prognostic value of the SI Index was not significantly modified by these clinical variables.

#### Mortality risk across sarcopenia stages: pre-sarcopenia, probable sarcopenia, and confirmed sarcopenia

Table 5 presents the association between distinct sarcopenia stages and all-cause mortality across the three cohorts. NHANES: confirmed sarcopenia HR 6.357 (95% CI 3.395–11.903, *p* < 0.001), probable sarcopenia HR 3.130 (95% CI 2.112–4.638, *p* < 0.001), pre-sarcopenia HR 2.184 (95% CI 1.118–4.267, *p* = 0.022). CHARLS: probable sarcopenia HR 2.459 and pre-sarcopenia HR 2.405, closely comparable, supporting their aggregation into the composite AMT category. C-OPTION: probable sarcopenia HR 3.671, confirmed sarcopenia HR 4.582; no mortality events were recorded among patients with pre-sarcopenia in this cohort, so the HR could not be estimated.

**Table 5:** Multivariable cox proportional hazards regression analysis for all-cause mortality according to sarcopenia status.

Factors		HR	95%CI	*p*-value
C-OPTION	Sarcopenia (*n*,%)	4.582	1.178–17.826	0.028
	Probable Sarcopenia (*n*,%)	3.671	1.553–8.675	0.003
	Pre-sarcopenia (*n*,%)	-	-	-
NHANES	Sarcopenia (*n*,%)	6.357	3.395–11.903	<0.001
	Probable Sarcopenia (*n*,%)	3.130	2.112–4.638	<0.001
	Pre-sarcopenia (*n*,%)	2.184	1.118–4.267	0.022
CHARLS	Sarcopenia (*n*,%)	5.306	3.363–8.370	<0.001
	Probable Sarcopenia (*n*,%)	2.459	1.934–3.127	<0.001
	Pre-sarcopenia (*n*,%)	2.405	1.322–4.377	0.004

Adjustments for Multivariate Models by Cohort: C-OPTION Cohort: Adjusted for UA, SCR, and SII. NHANES Cohort: Adjusted For History of Heart Disease and Stroke, GLU, DBP, eGFR, UACR, and SII. CHARLS Cohort: Adjusted for DBP, GLU, WBC, eGFR, gender, BMI, and CRP. BMI, Body Mass Index; CRP, C-Reactive Protein; DBP, Diastolic Blood Pressure; eGFR, Estimated Glomerular Filtration Rate; GLU, Blood Glucose; SCR, Serum Creatinine; SII, Systemic Immune-Inflammation Index; UA, Uric Acid; UACR, Urine Albumin-To-Creatinine Ratio; WBC, White Blood Cell Count.

## Discussion

In this prospective multi-cohort study encompassing 4,895 patients with CKD from three datasets (C-OPTION, NHANES, and CHARLS), we developed and validated a novel SI Index for predicting all-cause mortality. The SI Index, constructed as a weighted linear combination of sarcopenia status, AMT, and high inflammation (SI Index = 1.522 × Sarcopenia + 1.300 × Abnormal Muscle Traits + 0.880 × High Inflammation), demonstrated superior prognostic discrimination compared to its individual components, yielding C-indices ranging from 0.657 to 0.741 across cohorts. Incorporation of the SI Index into a base clinical model significantly enhanced predictive accuracy (improving C-indices from 0.565–0.726 to 0.729–0.768; all *p* < 0.001) and markedly improved model fit. Furthermore, this study revealed that probable sarcopenia and pre-sarcopenia already confer significant mortality risks, highlighting a continuum of risk that predates the onset of confirmed sarcopenia.

Our findings align with and significantly extend existing evidence regarding the prognostic roles of sarcopenia and inflammation in CKD. First, regarding the independent prognostic value of sarcopenia, we observed a markedly elevated mortality risk among sarcopenic patients (HR 2.184–6.357), a finding highly consistent with multiple large-scale cohort studies. A meta-analysis by Gomes et al. (33) of non-dialysis CKD patients showed that sarcopenia increased mortality risk by 143%. Wilkinson et al. (9), analyzing 8,767 CKD patients from the UK Biobank, found that sarcopenia reduced the 10-year survival rate by 4% (85% vs. 89%; HR 1.33, 95% CI: 1.07–1.66) and doubled the risk of end-stage renal disease (ESRD) (HR 1.98, 95% CI: 1.45–2.70). In dialysis populations, Kim et al. (11) reported an even higher mortality risk (HR 6.99, 95% CI: 1.84–26.58), underscoring the persistent prognostic significance of sarcopenia throughout CKD progression.

Second, high inflammatory status (assessed via SII or CRP) increased mortality risk by 43–141% (HR 1.43–2.41), consistent with prior literature: Guo et al. (34), using large-scale NHANES data, showed that elevated SII levels increased the risk of prevalent CKD (OR 1.37) and independently predicted a 15–48% increase in all-cause mortality (HR 1.15–1.48) (13); Goicoechea et al. (35) reported a 3.48-fold increase in mortality risk among patients with high CRP (>3 mg/L) (95% CI: 1.53–7.59); and Menon et al. (31), in a 10-year follow-up of the MDRD study, confirmed that CKD stage 3–4 patients with CRP ≥ 3.0 mg/L faced a 56% increased risk of all-cause mortality (HR 1.56) and a 94% increased risk of cardiovascular mortality (HR 1.94). Collectively, these data establish chronic inflammation as a potent predictor of mortality in CKD.

The superior predictive efficacy of the SI Index stems from its integration of two biologically intertwined yet mechanistically distinct pathological pathways in CKD—muscle wasting and systemic inflammation. Along the muscle-wasting pathway, accumulation of uremic toxins directly induces mitochondrial dysfunction and ATP depletion (36); metabolic acidosis accelerates protein catabolism via the glucocorticoid pathway and the ubiquitin-proteasome system (37); and the uremic milieu inhibits the IGF-1/Akt/mTOR signaling pathway, impeding protein synthesis (38), together creating a bidirectional imbalance of “accelerated breakdown + impaired synthesis.” Along the inflammatory pathway, pro-inflammatory cytokines (IL-6, TNF-*α*) promote muscle catabolism via NF-κB pathway activation (39), while also significantly increasing cardiovascular risk through endothelial dysfunction, atherosclerosis, and thrombosis (40); even in patients with preserved muscle function, high inflammatory status independently predicts mortality (HR 1.43–2.41 in this study), underscoring its value as an independent prognostic factor.

No significant interaction was observed between sarcopenia and inflammation (*p* > 0.05), suggesting that these two factors may influence prognosis through distinct mechanisms, exerting additive rather than synergistic effects. Although inflammation-induced muscle catabolism and uremic toxin-driven muscle wasting may interact to form a vicious cycle (36–39), their respective contributions to mortality risk appear to be largely independent and additive. This “dual burden” explains why the SI Index outperforms single indicators: assessing sarcopenia alone misses high-risk patients with high inflammation but preserved muscle, whereas assessing inflammation alone overlooks the risk in patients with low inflammation but severe muscle wasting; the SI Index quantifies the patient’s overall pathological burden by weighting and integrating these two dimensions.

The inclusion of the AMT category in the SI Index reflects an appreciation for the continuum of muscle decline. Although updated guidelines from EWGSOP2 (41) prioritize muscle strength, we retained the classification of ‘pre-sarcopenia’ (low muscle mass only) to dissect the specific contributions of muscle quantity versus quality to mortality risk—our results confirm that even this ‘preclinical’ stage independently predicts mortality, supporting the importance of monitoring muscle mass alongside muscle strength.

This study has three main strengths. First, the SI Index requires only routine clinical metrics (grip strength, height and weight, and complete blood count) for calculation, without the need for imaging or complex laboratory tests. Second, it employs a rigorous two-stage validation design: internal validation was first performed within the C-OPTION cohort via 10-fold cross-validation, followed by an assessment of cross-population generalizability in the NHANES and CHARLS cohorts, which differ substantially in population characteristics—enhancing confidence in the index’s transportability across real-world settings. Third, the SI Index demonstrates methodological flexibility, as its core lies in quantifying the pathological concept of “high inflammatory status” rather than depending on a specific biomarker: SII was used in the C-OPTION and NHANES cohorts and CRP in the CHARLS cohort, with both operationalized as a binary “high inflammation” indicator using clinically validated cutoffs (SII > 600; CRP > 3 mg/L). Because the comparison relies on this binarized status rather than the raw marker values themselves, the SI Index maintained robust predictive value across all three cohorts, further supporting its applicability across varied healthcare resource settings.

This study has the following limitations. First, the observational design limits causal inference; although extensive confounders were adjusted for, residual confounding cannot be excluded. Second, ASM/SMI were estimated using a simplified anthropometric equation (25), which is less accurate than DXA or BIA; in CKD patients, edema and volume overload may inflate body weight and SMI, leading to under-diagnosis of sarcopenia—future work should validate the index using direct measurement methods. Third, the discovery cohort had a limited sample size and number of events (only 45 deaths); the model used to derive the SI Index weights (Model 2) included five parameters, yielding an EPV of approximately 9, marginally below the conventional threshold of 10, with wide confidence intervals (e.g., HR for Sarcopenia, 95% CI 1.178–17.826), suggesting a risk of overfitting that warrants re-validation in larger cohorts. Fourth, NHANES and CHARLS are publicly available, population-representative datasets rather than dedicated CKD cohorts; their use here should be interpreted as a cross-population generalizability assessment rather than formal external validation. Fifth, neither dataset includes dialysis status, precluding cross-cohort comparison. Sixth, the median follow-up in C-OPTION was only 1.59 years, with the number at risk declining to 83 (15.4%) and 19 (3.5%) at years 5 and 7, respectively, and 37 of 45 deaths occurring within 5 years (Supplementary Table 7); the long-term AUC and calibration results should therefore be regarded as exploratory. Seventh, CKD ascertainment in NHANES relied on a single eGFR/UACR measurement, unable to confirm abnormalities persisting ≥ 3 months and thus carrying a risk of misclassification; this risk was partially mitigated in CHARLS via questionnaire-based corroboration, which was unavailable in NHANES.

## Conclusion

In summary, the SI Index developed and validated in this study provides a potent, reproducible, and clinically actionable novel tool for predicting all-cause mortality risk in CKD patients. By integrating two independent yet biologically intertwined pathological dimensions—muscle wasting and systemic inflammation—the SI Index achieves superior risk stratification compared to single markers (C-index 0.657–0.741) and demonstrates significant incremental prognostic value when added to models containing base clinical variables (improving C-index to 0.729–0.768). Its robust performance across three independent cohorts (covering 4,895 patients) and consistency across multiple subgroups confirm the generalizability and clinical utility of this index.

## Data Availability

The raw data supporting the conclusions of this article will be made available by the authors, without undue reservation.

## Glossary

AIC: Akaike information criterion
AMT: abnormal muscle traits
ASM: appendicular skeletal muscle mass
AUC: area under the curve
BIA: bioelectrical impedance analysis
BIC: Bayesian information criterion
BMI: body mass index
BUN: blood urea nitrogen
CHARLS: China Health and Retirement Longitudinal Study
CI: confidence interval
CKD: chronic kidney disease
C-OPTION: Chinese Observational Prospective Study of Ageing Population with Chronic Kidney Disease
CRP: C-reactive protein
DBP: diastolic blood pressure
DXA: dual-energy X-ray absorptiometry
eGFR: estimated glomerular filtration rate
ESKD: end-stage kidney disease
EWGSOP2: European Working Group on Sarcopenia in Older People 2
GLU: glucose
HB: hemoglobin
HDL: high-density lipoprotein cholesterol
HR: hazard ratio
IGF-1: insulin-like growth factor-1
LDL: low-density lipoprotein cholesterol
MICS: malnutrition-inflammation-cachexia syndrome
NHANES: National Health and Nutrition Examination Survey
PEW: protein-energy wasting
PLT: platelet count
ROC: receiver operating characteristic
RR: relative risk
SBP: systolic blood pressure
SCR: serum creatinine
SI Index: sarcopenia-inflammation index
SII: systemic immune-inflammation index
SMI: skeletal muscle mass index
TC: total cholesterol
TG: triglycerides
UA: uric acid
UACR: urinary albumin-to-creatinine ratio
UPS: ubiquitin-proteasome system
UTP: 24-h urinary total protein
VIF: variance inflation factor
WBC: white blood cell count
