# Supplementary material for: Development and multi-cohort validation of a sarcopenia-inflammation index for predicting all-cause mortality in patients with chronic kidney disease
Source: Front Nutr. 2026 Aug 24;13:1852039. doi: 10.3389/fnut.2026.1852039 (PMC13547068; doi:10.3389/fnut.2026.1852039)
Supplement: Supplementary file 1 [file Data_Sheet_1.docx]

**Supplementary Material**

# Development and Multi-cohort Validation of a Sarcopenia-Inflammation Index for Predicting All-cause Mortality in Patients with Chronic Kidney Disease

# Xiaolong Wang ^1^

# Jian Yang^1^

# Yanjun Liang^2^

# Zheyi Dong^1^

# Shuang Liang^1^

^1^Department of Nephrology, First Medical Center of Chinese PLA General Hospital, Chinese PLA Institute of Nephrology,. ^2^Department of Nephrology, Characteristic Medical Center of Chinese People's Armed Police Force, Tianjin, China.

Correspondence: Shuang Liang and Zheyi Dong

Department of Nephrology, Chinese PLA General Hospital, Chinese PLA Institute of Nephrology, State Key Laboratory of Kidney Diseases, National Clinical Research Center for Kidney Diseases, 28 Fuxing Road, Beijing 100853, China.

Tel +86 151 1024 6423.

Email: [liangshuang301@163.com](mailto:shengdai26@163.com;and)

**Supplementary Table 1** Possible SI Index Values by Combination of Muscle Status and Inflammation Status

| **Muscle Status** | **Inflammation Status** | **Calculation (β1 = AMT coefficient, β2 = Sarcopenia coefficient, β3 = High Inflammation coefficient)** | **SI Index Value** |
| --- | --- | --- | --- |
| Healthy (S1=0, S2=0) | Not high (I=0) | β1(0) + β2(0) + β3(0) | 0 |
| Healthy (S1=0, S2=0) | High (I=1) | β1(0) + β2(0) + β3(1) | β3 |
| AMT (S1=1, S2=0) | Not high (I=0) | β1(1) + β2(0) + β3(0) | β1 |
| AMT (S1=1, S2=0) | High (I=1) | β1(1) + β2(0) + β3(1) | β1 + β3 |
| Sarcopenia (S1=0, S2=1) | Not high (I=0) | β1(0) + β2(1) + β3(0) | β2 |
| Sarcopenia (S1=0, S2=1) | High (I=1) | β1(0) + β2(1) + β3(1) | β2 + β3 |

**Note:** β1, β2, and β3 denote the regression coefficients (log hazard ratios) for AMT, Sarcopenia, and High Inflammation, respectively, estimated from the multivariate Cox model in the discovery cohort (see Results, Section “Construction of the SI Index,” and Table 2, for the fitted coefficient values used in this study).

**Abbreviations:** AMT, Abnormal Muscle Traits.

**Supplementary Table 2** Baseline Characteristics of Patients with Chronic Kidney Disease (CKD) in the NHANES Cohort, Stratified by Survival Status

| Items | Survive (N = 1271) | Death (N = 460) | Total (N = 1731) | P-value |
| --- | --- | --- | --- | --- |
| Age (years) | 57.00 (42.00, 70.00) | 77.00 (67.00, 80.00) | 63.00 (47.00, 75.00) | <0.001 |
| Sex (male) | 623 (49.0%) | 260 (56.5%) | 883 (51.0%) | 0.007 |
| Height (cm) | 166.00 (158.70, 173.20) | 165.25 (157.00, 172.60) | 165.80 (158.50, 172.90) | 0.081 |
| Weight (kg) | 79.50 (67.70, 95.60) | 76.95 (64.40, 94.10) | 78.80 (66.60, 95.50) | 0.012 |
| BMI | 29.00 (24.80, 34.00) | 28.15 (24.20, 32.90) | 28.70 (24.70, 33.70) | 0.042 |
| SBP (mmHg) | 128.67 (116.00, 144.00) | 135.33 (120.67, 154.67) | 130.67 (116.67, 147.33) | <0.001 |
| DBP (mmHg) | 71.33 (62.67, 80.00) | 65.33 (54.67, 74.00) | 70.00 (60.67, 78.67) | <0.001 |
| Grip-max (kg) | 31.80 (25.10, 40.00) | 25.40 (18.90, 32.50) | 29.90 (23.50, 38.20) | <0.001 |
| Low-grip (n, %) | 67 (5.3%) | 132 (28.7%) | 199 (11.5%) | <0.001 |
| SMI (kg/m²) | 8.09 (7.08, 9.16) | 7.78 (6.83, 8.98) | 8.02 (7.01, 9.12) | 0.002 |
| Low-SMI (n, %) | 62 (4.9%) | 66 (14.3%) | 128 (7.4%) | <0.001 |
| Laboratory examination |  |  |  |  |
| HB (g/L) | 137.00 (126.00, 147.00) | 131.00 (119.00, 142.00) | 135.00 (125.00, 146.00) | <0.001 |
| WBC (10^9/L) | 7.10 (5.90, 8.60) | 7.20 (5.90, 8.60) | 7.10 (5.90, 8.60) | 0.594 |
| Neutrophils (%) | 60.20 (53.50, 66.20) | 63.10 (56.80, 70.10) | 61.00 (54.20, 67.10) | <0.001 |
| Lymphocytes (%) | 28.30 (23.00, 34.30) | 23.80 (18.30, 30.15) | 27.10 (21.70, 33.40) | <0.001 |
| PLT (10^9/L) | 228.00 (192.00, 273.00) | 207.00 (167.00, 253.00) | 222.00 (185.00, 269.00) | <0.001 |
| TC (mmol/L) | 4.70(4.05, 5.58) | 4.52 (3.82, 5.43) | 4.65 (4.00, 5.53) | 0.003 |
| TG (mmol/L) | 1.25 (0.84, 1.84) | 1.27 (0.95, 1.91) | 1.25 (0.87, 1.86) | 0.257 |
| HDL (mmol/L) | 1.27 (1.03,1.52) | 1.21 (1.01, 1.52) | 1.24 (1.03, 1.52) | 0.134 |
| LDL (mmol/L) | 2.70 (2.13, 3.39) | 2.48 (1.95, 3.29) | 2.66 (2.09, 3.37) | 0.028 |
| GLU (mmol/L) | 5.78 (5.22, 6.89) | 6.22 (5.55, 8.05) | 5.92 (5.33, 7.11) | <0.001 |
| BUN (mmol/L) | 5.00 (3.57, 6.78) | 7.50 (5.00, 10.00) | 5.35 (3.92, 7.50) | <0.001 |
| UA (umol/L) | 339.15 (257.14, 416.50) | 368.90 (303.45, 452.20) | 351.05(286.60, 428.40) | <0.001 |
| SCR (umol/L) | 81.23 (64.45, 116.56) | 113.02 (82.11, 141.28) | 88.3 (67.10, 122.74) | <0.001 |
| eGFR (mL/min/1.73m²) | 94.34 (57.99, 114.63) | 58.21 (44.84, 86.34) | 85.20 (54.53, 109.05) | <0.001 |
| UACR (mg/g) | 52.50 (37.73, 118.19) | 59.66 (30.58, 190.72) | 53.46 (33.02, 126.15) | 0.086 |
| CKD Stage (n, %) |  |  |  | <0.001 |
| G1 (≥90 mL/min/1.73m²) | 660 (54.7%) | 87 (20.3%) | 747 (45.7%) |  |
| G2 (60–89) | 201 (16.7%) | 103 (24.1%) | 304 (18.6%) |  |
| G3a (45–59) | 230 (19.1%) | 130 (30.4%) | 360 (22.0%) |  |
| G3b (30–44) | 75 (6.2%) | 71 (16.6%) | 146 (8.9%) |  |
| G4 (15–29) | 23 (1.9%) | 18 (4.2%) | 41 (2.5%) |  |
| G5 (<15) | 17 (1.4%) | 19 (4.4%) | 36 (2.2%) |  |
| SII | 485.35 (328.89, 700.84) | 546.86 (355.59, 833.77) | 497.40 (335.61, 737.48) | <0.001 |
| High Inflammation (n, %) | 446 (35.1%) | 208 (45.2%) | 654 (37.8%) | <0.001 |
| Comorbidities |  |  |  |  |
| Hypertension (n, %) | 702 (55.2%) | 355 (77.2%) | 1,057 (61.1%) | <0.001 |
| Diabetes (n, %) | 341 (26.8%) | 167 (36.3%) | 508 (29.3%) | <0.001 |
| Heart disease (n, %) | 170 (13.4%) | 160 (34.8%) | 330 (19.1%) | <0.001 |
| Stroke (n, %) | 75 (5.9%) | 86 (18.7%) | 161 (9.3%) | <0.001 |
| Medicine |  |  |  |  |
| Antihypertensive Drug (n, %) | 624 (49.1%) | 241 (52.4%) | 865 (50.0%) | 0.247 |
| Antidiabetic Drug (n, %) | 309 (24.3%) | 163 (35.4%) | 472 (27.3%) | <0.001 |
| Lipid-lowering Drug (n, %) | 409 (32.2%) | 240 (52.2%) | 649 (37.5%) | <0.001 |
| Smoking (n, %) | 590 (46.4%) | 263 (57.2%) | 853 (49.3%) | <0.001 |
| Drinking (n, %) | 1,150 (90.5%) | 428 (93.0%) | 1,578 (91.2%) | 0.118 |
| Sarcopenia Status (n, %) |  |  |  | <0.001 |
| Healthy (n, %) | 1,154 (90.8%) | 294 (63.9%) | 1,448 (83.7%) |  |
| Abnormal Muscle Traits (n, %) | 105 (8.3%) | 134 (29.1%) | 239 (13.8%) |  |
| Sarcopenia (n, %) | 12 (0.9%) | 32 (7.0%) | 44 (2.5%) |  |
| Follow-time (years) | 6.83 (5.92,7.92) | 3.58 (2.08, 5.31) | 6.42 (5.25, 7.58) | <0.001 |

**Abbreviations:** BMI, Body Mass Index; BUN, Blood Urea Nitrogen; CKD, Chronic Kidney Disease; DBP, Diastolic Blood Pressure; eGFR, estimated Glomerular Filtration Rate; GLU, Glucose; HB, Hemoglobin; HDL, High-Density Lipoprotein Cholesterol; LDL, Low-Density Lipoprotein Cholesterol; PLT, Platelet Count; SBP, Systolic Blood Pressure; SCR, Serum Creatinine; SII, Systemic Immune-Inflammation Index; SMI, Skeletal Muscle Index; TC, Total Cholesterol; TG, Triglycerides; UA, Uric Acid; UTP, 24-hour Urinary Total Protein; WBC, White Blood Cell Count; Grip-max, Maximum Grip Strength; NHANES, National Health and Nutrition Examination Survey.

**Supplementary Table 3** Baseline Characteristics of Patients with Chronic Kidney Disease (CKD) in the CHARLS Cohort, Grouped by Survival Status

| Items | Survive (N = 2258) | Death (N = 367) | Total (N =2625) | P-value |
| --- | --- | --- | --- | --- |
| Age (years) | 60.00 (52.00, 67.00) | 72.00 (65.00, 78.00) | 61.00 (53.00, 69.00) | <0.001 |
| Sex (male) | 1,160 (51.4%) | 250 (68.1%) | 1,410 (53.7%) | <0.001 |
| Height (cm) | 158.00 (152.00, 164.00) | 158.00 (151.00, 164.00) | 158.00 (152.00, 164.00) | 0.608 |
| Weight (kg) | 57.40 (51.30, 65.30) | 53.90 (46.00, 62.50) | 57.00 (50.60, 65.10) | <0.001 |
| BMI | 23.15 (20.79, 25.93) | 21.63 (19.35, 24.48) | 22.97 (20.51, 25.75) | <0.001 |
| SBP (mmHg) | 126.00 (114.33, 141.00) | 138.33 (122.67, 156.00) | 127.33 (115.33, 143.00) | <0.001 |
| DBP (mmHg) | 75.33 (67.67, 82.67) | 77.67 (69.00, 86.00) | 75.33 (67.67, 83.33) | 0.002 |
| Grip-max (kg) | 29.50 (22.50, 36.00) | 25.00 (18.40, 32.00) | 29.00 (22.00, 36.00) | <0.001 |
| Low-grip (n, %) | 380 (16.8%) | 166 (45.2%) | 546 (20.8%) | <0.001 |
| SMI (kg/m²) | 8.38 (7.12, 9.37) | 8.20 (6.88, 9.10) | 8.36 (7.09, 9.32) | 0.005 |
| Low-SMI (n, %) | 123 (5.5%) | 55 (15.2%) | 178 (6.8%) | <0.001 |
| Laboratory examination |  |  |  |  |
| HB (g/L) | 132.00 (115.00, 146.00) | 127.00 (109.00, 145.00) | 131.00 (113.00, 146.00) | 0.014 |
| WBC (10^9/L) | 5.80 (4.80, 7.00) | 6.20 (5.10, 7.87) | 5.80 (4.80, 7.10) | <0.001 |
| PLT (10^9/L) | 205.00 (158.00, 255.00) | 199.00 (158.00, 255.00) | 204.00 (158.00, 255.00) | 0.689 |
| CRP (mg/dL) | 1.37 (0.72, 2.63) | 1.90 (0.90, 4.40) | 1.40 (0.74, 2.80) | <0.001 |
| TC (mmol/L) | 4.75 (4.19, 5.39) | 4.71 (4.14, 5.50) | 4.75 (4.18, 5.41) | 0.958 |
| TG (mmol/L) | 1.25 (0.89, 1.86) | 1.23 (0.86, 1.83) | 1.25 (0.89, 1.85) | 0.637 |
| HDL (mmol/L) | 1.27 (1.08, 1.49) | 1.28 (1.04, 1.48) | 1.27 (1.07, 1.49) | 0.525 |
| LDL (mmol/L) | 2.68 (2.20, 3.24) | 2.70 (2.18, 3.23) | 2.69 (2.19, 3.24) | 0.875 |
| GLU (mmol/L) | 5.40 (5.00, 6.00) | 5.60 (5.10, 6.93) | 5.40 (5.05, 6.11) | <0.001 |
| BUN (mmol/L) | 5.80 (4.70, 7.24) | 6.82 (5.39, 8.67) | 5.98 (4.80, 7.41) | <0.001 |
| UA (umol/L) | 303.35 (243.87, 380.67) | 350.93 (275.99, 433.43) | 309.30 (248.63, 388.40) | <0.001 |
| SCR (umol/L) | 79.83 (63.86, 118.86) | 109.10 (78.94, 130.39) | 84.27 (65.64, 118.86) | <0.001 |
| eGFR (mL/min/1.73m²) | 89.34 (57.08, 103.04) | 57.78 (47.48, 82.55) | 85.86 (55.04, 101.57) | <0.001 |
| CKD Stage (n, %) |  |  |  | **<**0.001 |
| G1 (≥90 mL/min/1.73m²) | 1,105 (48.9%) | 67 (18.3%) | 1,172 (44.6%) |  |
| G2 (60–89) | 462 (20.5%) | 80 (21.8%) | 542 (20.6%) |  |
| G3a (45–59) | 372 (16.5%) | 147 (40.1%) | 519 (19.8%) |  |
| G3b (30–44) | 278 (12.3%) | 51 (13.9%) | 329 (12.5%) |  |
| G4 (15–29) | 39 (1.7%) | 16 (4.4%) | 55 (2.1%) |  |
| G5 (<15) | 2 (0.1%) | 6 (1.6%) | 8 (0.3%) |  |
| High Inflammation (n, %) | 476 (21.1%) | 123 (33.5%) | 599 (22.8%) | <0.001 |
| Comorbidities |  |  |  |  |
| Hypertension (n, %) | 787 (34.9%) | 181 (49.3%) | 968 (36.9%) | <0.001 |
| Diabetes (n, %) | 259 (11.5%) | 67 (18.3%) | 326 (12.4%) | <0.001 |
| Heart disease (n, %) | 501 (22.2%) | 98 (26.7%) | 599 (22.8%) | 0.065 |
| Stroke (n, %) | 121 (5.4%) | 25 (6.8%) | 146 (5.6%) | 0.315 |
| Medicine |  |  |  |  |
| Antihypertensive Drug (n, %) | 585 (25.9%) | 133 (36.2%) | 718 (27.4%) | <0.001 |
| Antidiabetic Drug (n, %) | 176 (7.8%) | 48 (13.1%) | 224 (8.5%) | 0.001 |
| Lipid-lowering Drug (n, %) | 225 (10.0%) | 35 (9.5%) | 260 (9.9%) | 0.873 |
| Smoking (n, %) | 954 (42.2%) | 205 (56.0%) | 1,159 (44.2%) | <0.001 |
| Drinking (n, %) | 946 (41.9%) | 179 (48.9%) | 1,125 (42.9%) | 0.013 |
| Sarcopenia Status (n, %) |  |  |  | <0.001 |
| Healthy (n, %) | 1,721 (76.2%) | 178 (48.5%) | 1,899 (72.3%) |  |
| Abnormal Muscle Traits (n, %) | 489 (21.7%) | 149 (40.6%) | 638 (24.3%) |  |
| Sarcopenia (n, %) | 48 (2.1%) | 40 (10.9%) | 88 (3.4%) |  |
| Follow-time (years) | 5.00 (5.00, 9.00) | 3.00 (1.00, 5.00) | 5.00 (5.00, 9.00) | <0.001 |

**Abbreviations:** BMI, Body Mass Index; BUN, Blood Urea Nitrogen; CHARLS, China Health and Retirement Longitudinal Study; CKD, Chronic Kidney Disease; CRP, C-Reactive Protein; DBP, Diastolic Blood Pressure; eGFR, estimated Glomerular Filtration Rate; GLU, Glucose; Grip-max, Maximum Grip Strength; HB, Hemoglobin; HDL, High-Density Lipoprotein; LDL, Low-Density Lipoprotein; PLT, Platelet Count; SBP, Systolic Blood Pressure; SCR, Serum Creatinine; SMI, Skeletal Muscle Index; TC, Total Cholesterol; TG, Triglycerides; UA, Uric Acid; WBC, White Blood Cell.

**Supplementary Table 4** Comprehensive Comparison of Baseline Characteristics Across the C-OPTION, NHANES, and CHARLS Cohorts

| Items | C-OPTION | NHANES | CHARLS | P-value |
| --- | --- | --- | --- | --- |
| Age (years) | 68.00 (64.00, 74.00) | 63.00 (47.00, 75.00) | 61.00 (53.00, 69.00) | <0.001 |
| Sex (male) | 342 (64.0%) | 883 (51.0%) | 1410 (53.7%) | <0.001 |
| Height (cm) | 165.00 (159.00, 171.00) | 165.80 (158.50, 172.90) | 158.00 (152.00, 164.00) | <0.001 |
| Weight (kg) | 68.00 (61.00, 75.90) | 78.80 (66.60, 95.50) | 57.00 (50.60, 65.10) | <0.001 |
| BMI | 24.80 (22.66, 27.34) | 28.70 (24.70, 33.70) | 22.97 (20.51, 25.75) | <0.001 |
| SBP (mmHg) | 137.00 (125.25, 148.50) | 130.67 (116.67, 147.33) | 127.33 (115.33, 143.00) | <0.001 |
| DBP (mmHg) | 79.50 (73.50, 87.50) | 70.00 (60.67, 78.67) | 75.33 (67.67, 83.33) | <0.001 |
| Grip-max (kg) | 27.00 (20.60, 35.80) | 29.90 (23.50, 38.20) | 29.00 (22.00, 36.00) | <0.001 |
| Low-grip (n, %) | 182 (34.1%) | 199 (11.5%) | 546 (20.8%) | <0.001 |
| SMI (kg/m²) | 7.42 (6.40, 8.05) | 8.02 (7.01, 9.12) | 8.36 (7.09, 9.32) | <0.001 |
| Low-SMI (n, %) | 36(6.7%) | 128 (7.4%) | 178 (6.8%) | <0.001 |
| Laboratory examination |  |  |  |  |
| HB (g/L) | 120.00 (105.50, 133.00) | 135.00 (125.00, 146.00) | 131.00 (113.00, 146.00) | <0.001 |
| WBC (10^9/L) | 6.45 (5.33, 7.90) | 7.10 (5.90, 8.60) | 5.80 (4.80, 7.10) | <0.001 |
| PLT (10^9/L) | 204.00 (167.00, 248.00) | 222.00 (185.00, 269.00) | 204.00 (158.00, 255.00) | <0.001 |
| TC (mmol/L) | 4.83 (3.94, 6.14) | 4.65 (4.00, 5.53) | 4.75 (4.18, 5.41) | 0.003 |
| TG (mmol/L) | 1.68 (1.17, 2.45) | 1.25 (0.87, 1.86) | 1.25 (0.89, 1.85) | <0.001 |
| HDL (mmol/L) | 1.15 (0.97, 1.42) | 1.24 (1.03, 1.52) | 1.27 (1.07, 1.49) | <0.001 |
| LDL (mmol/L) | 2.91 (2.21, 3.87) | 2.66 (2.09, 3.37) | 2.69 (2.19, 3.24) | <0.001 |
| GLU (mmol/L) | 5.13 (4.54, 6.12) | 5.92 (5.33, 7.11) | 5.40 (5.05, 6.11) | <0.001 |
| BUN (mmol/L) | 8.60 (6.41, 12.51) | 5.35 (3.92, 7.50) | 5.98 (4.80, 7.41) | <0.001 |
| UA (umol/L) | 398.00 (333.40, 470.00) | 351.05(286.60, 428.40) | 309.30 (248.63, 388.40) | <0.001 |
| SCR (umol/L) | 121.50 (81.00, 180.15) | 88.3 (67.10, 122.74) | 84.27 (65.64, 118.86) | <0.001 |
| eGFR (mL/min/1.73m²) | 48.81 (27.38, 76.94) | 85.20 (54.53, 109.05) | 85.86 (55.04, 101.57) | <0.001 |
| CKD Stage (n, %) |  |  |  | <0.001 |
| G1 (≥90 mL/min/1.73m²) | 87 (16.4%) | 747 (45.7%) | 1,172 (44.6%) |  |
| G2 (60–89) | 106 (20.0%) | 304 (18.6%) | 542 (20.6%) |  |
| G3a (45–59) | 92 (17.3%) | 360 (22.0%) | 519 (19.8%) |  |
| G3b (30–44) | 95 (17.9%) | 146 (8.9%) | 329 (12.5%) |  |
| G4 (15–29) | 124 (23.4%) | 41 (2.5%) | 55 (2.1%) |  |
| G5 (<15) | 27 (5.1%) | 36 (2.2%) | 8 (0.3%) |  |
| High Inflammation (n, %) | 183 (34.5%) | 654 (37.8%) | 599 (22.8%) | <0.001 |
| Comorbidities |  |  |  |  |
| Hypertension (n, %) | 420 (77.9%) | 1057 (61.1%) | 968 (36.9%) | <0.001 |
| Diabetes (n, %) | 191 (35.4%) | 508 (29.3%) | 326 (12.4%) | <0.001 |
| Heart disease (n, %) | 97 (18.0%) | 330 (19.1%) | 599 (22.8%) | 0.002 |
| Stroke (n, %) | 101 (18.7%) | 161 (9.6%) | 146 (5.6%) | <0.001 |
| Medicine |  |  |  |  |
| Antihypertensive Drug (n, %) | 435 (80.7%) | 865 (50.0%) | 718 (27.4%) | <0.001 |
| Antidiabetic Drug (n, %) | 167 (31.0%) | 472 (27.3%) | 224 (8.5%) | <0.001 |
| Lipid-lowering Drug (n, %) | 219 (40.6%) | 649 (37.5%) | 260 (9.9%) | <0.001 |
| Smoking (n, %) | 130 (38.0%) | 853 (49.3%) | 1159 (44.2%) | <0.001 |
| Drinking (n, %) | 108 (31.6%) | 1578 (91.2%) | 1125 (42.9%) | <0.001 |
| Sarcopenia Status (n, %) |  |  |  | <0.001 |
| Healthy (n, %) | 357 (66.2%) | 1448 (83.7%) | 1899 (72.3%) |  |
| Abnormal Muscle Traits (n, %) | 150 (27.8%) | 239 (13.8%) | 638 (24.3%) |  |
| Sarcopenia (n, %) | 32 (5.9%) | 44 (2.5%) | 88 (3.4%) |  |

**Abbreviations:** BMI, Body Mass Index; SBP/DBP, Systolic/Diastolic Blood Pressure; SMI, Skeletal Muscle Index; HB, Hemoglobin; WBC, White Blood Cell Count; PLT, Platelet Count; TC, Total Cholesterol; TG, Triglycerides; HDL/LDL, High/Low-Density Lipoprotein; GLU, Glucose; BUN, Blood Urea Nitrogen; UA, Uric Acid; SCR, Serum Creatinine; eGFR, estimated Glomerular Filtration Rate.

**Supplementary Table 5** Multivariate Cox Regression Analysis of Sarcopenia Status, Inflammation, and Associated Factors with All-Cause Mortality in the NHANES Cohort

| **Factors** | **HR** | **95%CI** | **P-value** |
| --- | --- | --- | --- |
| Sarcopenia (n,%) | 5.947 | 3.405-11.928 | <0.001 |
| Abnormal Muscle Traits(n,%) | 2.862 | 1.999-4.099 | <0.001 |
| High inflammation (n,%) | 1.494 | 1.093-2.043 | 0.012 |
| Heart disease (n,%) | 1.915 | 1.370-2.678 | <0.001 |
| Stroke (n,%) | 1.952 | 1.297-2.939 | 0.003 |
| GLU (mmol/L) | 1.004 | 1.000-1.008 | 0.040 |
| DBP (mmHg) | 0.988 | 0.978-0.998 | 0.024 |
| eGFR (mL/min/1.73m²) | 0.990 | 0.983-0.996 | 0.003 |
| UACR (mg/g) | 1.000 | 1.000-1.000 | 0.016 |

**Abbreviations:** HR: Hazard Ratio , 95% CI: 95% Confidence Interval , P-value: Probability value , GLU: Glucose, DBP: Diastolic Blood Pressure, eGFR: estimated Glomerular Filtration Rate, UACR: Urinary Albumin-to-Creatinine Ratio.

**Supplementary Table 6** Multivariate Cox Regression Analysis of Sarcopenia Status, Inflammation, and Associated Factors with All-Cause Mortality in the CHARLS Cohort

| **Factors** | **HR** | **95%CI** | **P-value** |
| --- | --- | --- | --- |
| Sarcopenia (n,%) | 5.872 | 3.766-9.156 | 0.001 |
| Abnormal Muscle Traits(n,%) | 2.464 | 1.961-3.095 | 0.001 |
| High inflammation (n,%) | 1.425 | 1.131-1.796 | 0.003 |
| DBP (mmHg) | 1.016 | 1.007-1.025 | 0.001 |
| GLU (mmol/L) | 1.003 | 1.000-1.005 | 0.019 |
| WBC (10^9/L) | 1.057 | 1.003-1.114 | 0.039 |
| eGFR (mL/min/1.73 m^2^) | 0.983 | 0.977-0.989 | < 0.001 |
| Gender (female) | 0.441 | 0.318-0.61 | < 0.001 |
| BMI | 0.917 | 0.888-0.947 | < 0.001 |

**Abbreviations:** HR: Hazard Ratio, 95% CI: 95% Confidence Interval, P-value: Probability value, DBP: Diastolic Blood Pressure, GLU: Glucose, WBC: White Blood Cell, eGFR: estimated Glomerular Filtration Rate, BMI: Body Mass Index.

**Supplementary Table 7** Number at Risk and Cumulative Events by Follow-up Time in the C-OPTION Cohort

| Follow-up Time | N at Risk, n (%) | Cumulative Deaths, n |
| --- | --- | --- |
| 1 year | 371 (68.8%) | 19 |
| 3 years | 151 (28.0%) | 29 |
| 5 years | 83 (15.4%) | 37 |
| 7 years | 19 (3.5%) | 45 |

Note: N at risk denotes the number of C-OPTION participants (total N = 539) with follow-up time ≥ the specified time point (i.e., still under observation, event-free or censored, at that time). Cumulative deaths denotes the total number of death events that had occurred by the specified time point (of 45 deaths overall; median follow-up in this cohort was 1.59 years). The 9-year time point was not estimable due to insufficient follow-up and is therefore not reported for this cohort in Table 3.

**Supplementary Figure 1** Construction of the Sarcopenia-Inflammation Index

**Supplementary Figure 2** Kaplan-Meier survival curves for all-cause mortality stratified by SII levels in the NHANES cohort

**Abbreviations:** SII, Systemic Immune-Inflammation Index

**Supplementary Figure 3** Kaplan-Meier survival analysis according to sarcopenia status in the NHANES cohort

**Abbreviations:** AMT, Abnormal Muscle Traits

**Supplementary Figure 4** Kaplan-Meier survival curves for all-cause mortality stratified by CRP levels in the CHARLS cohort

**Abbreviation:** CRP, C-reactive protein

**Supplementary Figure 5** Kaplan-Meier survival analysis according to sarcopenia status in the CHARLS cohort

**Abbreviations:** AMT, Abnormal Muscle Traits

**Supplementary Figure 6** Univariate Cox Regression Analysis of Risk Factors for All-Cause Mortality in the NHANES cohort

**Abbreviations:** BMI, body mass index; CI, confidence interval; DBP, diastolic blood pressure; eGFR, estimated glomerular filtration rate; HDL, high-density lipoprotein; HR, hazard ratio; LDL, low-density lipoprotein; NHANES, National Health and Nutrition Examination Survey; SBP, systolic blood pressure; SII, systemic immune-inflammation index; UACR, urinary albumin-to-creatinine ratio.

**Supplementary Figure 7** Univariate Cox Regression Analysis of Risk Factors for All-Cause Mortality in the CHARLS cohort

**Abbreviations:** BMI, body mass index;DBP,diastolic blood pressure; CI, confidence interval; CRP, C-reactive protein; CHARLS, China Health and Retirement Longitudinal Study; eGFR, estimated glomerular filtration rate; HDL, high-density lipoprotein; HR, hazard ratio; LDL, low-density lipoprotein

**Supplementary Figure 8** Time-dependent ROC curves illustrating the predictive performance of the SI Index, Sarcopenia status, and SII Group for mortality in the NHANES cohort at 1, 3, 5, and 7 years.

**Abbreviations:** SII, Systemic Immune-Inflammation Index; SI Index, Sarcopenia-Inflammation Index

**Supplementary Figure 9** Time-dependent ROC curves illustrating the predictive performance of the SI Index, Sarcopenia status, and SII Group for mortality in the CHARLS cohort at 3, 5, 7 and 9 years.

**Abbreviations:** CRP, C-reactive protein; SI Index, Sarcopenia-Inflammation Index

**Supplementary Figure 10** Comparison of the concordance index (C-index) for the SI Index, Sarcopenia status, and SII Group in the NHANES cohort

**Abbreviations:** SII, Systemic Immune-Inflammation Index; SI Index, Sarcopenia-Inflammation Index

**Supplementary Figure 11** Comparison of the concordance index (C-index) for the SI Index, Sarcopenia status, and SII Group in the CHARLS cohort

**Abbreviations:** CRP, C-reactive protein; SI Index, Sarcopenia-Inflammation Index

**Supplementary Figure 12** Time-dependent ROC analysis comparing the predictive performance of the Base model and the Combined model for all-cause mortality in the NHANES cohort.

Note: (A) 1-year, (B) 3-year, (C) 5-year, and (D) 7-year ROC curves. The blue line represents the Base model, and the red line represents the Combined model. The Base models were adjusted for history of stroke and heart disease,GLU, DBP, UACR, and eGFR; The Combined model was Base model incorporating the SI Index.

**Abbreviations:** GLU, blood glucose; DBP, diastolic blood pressure; UACR, urine albumin-to-creatinine ratio; eGFR, estimated glomerular filtration rate; NHANES, National Health and Nutrition Examination Survey; SI Index, Sarcopenia-Inflammation Index.

**Supplementary Figure 13** Time-dependent ROC analysis comparing the predictive performance of the Base model and the Combined model for all-cause mortality in the CHARLS cohort.

Note: (A) 3-year, (B) 5-year, (C) 7-year, and (D) 9-year ROC curves. The blue line represents the Base model, and the red line represents the Combined model. The Base models were adjusted for DBP, WBC, GLU, BMI, eGFR, and Gender; The Combined model was Base model incorporating the SI Index.

**Abbreviations:** CHARLS, China Health and Retirement Longitudinal Study; GLU, blood glucose; DBP, diastolic blood pressure; WBC, white blood cell count; eGFR, estimated glomerular filtration rate; SI Index, Sarcopenia-Inflammation Index.

**Supplementary Figure 14** Internal validation of predictive performance comparing the Base model and Combined model using 10-fold cross-validation in the C-OPTION cohort

Note: The boxplots illustrate the distribution of Concordance Index (C-index) values derived from 10-fold cross-validation. The Base model (green) includes uric acid, and serum creatinine, while the Combined model (orange) additionally incorporates the SI Index. The red diamonds represent the mean C-index, and the horizontal lines within the boxes indicate the median values. The scatter points represent the C-index obtained for each individual fold. The Base models were adjusted for UA and SCR; The Combined model was Base model incorporating the SI Index.

**Abbreviations:** C-OPTION, Chinese Observational Prospective Study of Ageing Population with Chronic Kidney Disease; SI Index, Sarcopenia-Inflammation Index

**Supplementary Figure 15** Internal validation of predictive performance comparing the Base model and Combined model using 10-fold cross-validation in the NHANES cohort

Note: The boxplots illustrate the distribution of Concordance Index (C-index) values derived from 10-fold cross-validation. The Base model (green) includes uric acid, and serum creatinine, while the Combined model (orange) additionally incorporates the SI Index. The red diamonds represent the mean C-index, and the horizontal lines within the boxes indicate the median values. The scatter points represent the C-index obtained for each individual fold. The Base models were adjusted for history of stroke and heart disease,GLU, DBP, UACR, and eGFR; The Combined model was Base model incorporating the SI Index.

**Abbreviations:** GLU, blood glucose; DBP, diastolic blood pressure; UACR, urine albumin-to-creatinine ratio; eGFR, estimated glomerular filtration rate; NHANES, National Health and Nutrition Examination Survey; SI Index, Sarcopenia-Inflammation Index.

**Supplementary Figure 16** Internal validation of predictive performance comparing the Base model and Combined model using 10-fold cross-validation in the CHARLS cohort

Note: The boxplots illustrate the distribution of Concordance Index (C-index) values derived from 10-fold cross-validation. The Base model (green) includes uric acid, and serum creatinine, while the Combined model (orange) additionally incorporates the SI Index. The red diamonds represent the mean C-index, and the horizontal lines within the boxes indicate the median values. The scatter points represent the C-index obtained for each individual fold. The Base models were adjusted for DBP, WBC, GLU, BMI, eGFR, and Gender; The Combined model was Base model incorporating the SI Index.

**Abbreviations:** CHARLS, China Health and Retirement Longitudinal Study; GLU, blood glucose; DBP, diastolic blood pressure; WBC, white blood cell count; eGFR, estimated glomerular filtration rate; SI Index, Sarcopenia-Inflammation Index.

**Supplementary Figure 17** Calibration curves comparing the predictive accuracy of the Base Model and the Combined Model for all-cause mortality in the C-OPTION cohort

Note: The blue circles and lines represent the Base Model (adjusted for uric acid and serum creatinine). The green triangles and lines represent the Combined Model (incorporating the SI Index into the Base Model). Shaded areas represent 95% confidence intervals.

**Abbreviations:** C-OPTION, Chinese Observational Prospective Study of Ageing Population with Chronic Kidney Disease; SI Index, Sarcopenia-Inflammation Index

**Supplementary Figure 18** Calibration curves comparing the predictive accuracy of the Base Model and the Combined Model for all-cause mortality in the NHANES cohort

Note: The blue circles and lines represent the Base Model (adjusted for history of stroke and heart disease,GLU, DBP, UACR, and eGFR). The green triangles and lines represent the Combined Model (incorporating the SI Index into the Base Model). Shaded areas represent 95% confidence intervals.

**Abbreviations:** GLU, blood glucose; DBP, diastolic blood pressure; UACR, urine albumin-to-creatinine ratio; eGFR, estimated glomerular filtration rate; NHANES, National Health and Nutrition Examination Survey; SI Index, Sarcopenia-Inflammation Index.

**Supplementary Figure 19** Calibration curves comparing the predictive accuracy of the Base Model and the Combined Model for all-cause mortality in the CHARLS cohort

Note: The blue circles and lines represent the Base Model (adjusted for DBP, WBC, GLU, BMI, eGFR, and Gender). The green triangles and lines represent the Combined Model (incorporating the SI Index into the Base Model). Shaded areas represent 95% confidence intervals.

**Abbreviations:** CHARLS, China Health and Retirement Longitudinal Study; GLU, blood glucose; DBP, diastolic blood pressure; WBC, white blood cell count; eGFR, estimated glomerular filtration rate; SI Index, Sarcopenia-Inflammation Index.
